# Fractional order cancer model infection in human with CD8+ T cells and anti-PD-L1 therapy: simulations and control strategy

**DOI:** 10.1038/s41598-024-66593-x

**Published:** 2024-07-15

**Authors:** Kottakkaran Sooppy Nisar, Muhammad Owais Kulachi, Aqeel Ahmad, Muhammad Farman, Muhammad Saqib, Muhammad Umer Saleem

**Affiliations:** 1 Department of Mathematics, College of Science and Humanities in Alkharj, Prince Sattam Bin Abdulaziz University, 11942 Alkharj, Saudi Arabia; 2 Saveetha School of Engineering, SIMATS, Chennai, India; 3 Department of Mathematics, Ghazi University, Dera Ghazi Khan, 32200 Pakistan; 4 Department of Mathematics, Mathematics Research Center, Near East University, Near East Boulevard, 99138 Nicosia, North Cyprus Turkey; 5 Department of Computer Science and Mathematics, Lebanese American University, Beirut, Lebanon; 6 Institute of Mathematics, Khawaja Fareed University of Engineering and Information Technology, Rahim Yar Khan, 64200 Pakistan; 7 Department of Mathematics, University of Education, Lahore, 54100 Pakistan

**Keywords:** Mathematical modeling, Cancer model, Fractional operator, Stability analysis, Boundedness, Global derivative, Cancer, Diseases, Mathematics and computing

## Abstract

In order to comprehend the dynamics of disease propagation within a society, mathematical formulations are essential. The purpose of this work is to investigate the diagnosis and treatment of lung cancer in persons with weakened immune systems by introducing cytokines ($IL_{2}  \&  IL_{12}$) and anti-PD-L1 inhibitors. To find the stable position of a recently built system TCD$IL_{2} IL_{12}$Z, a qualitative and quantitative analysis are taken under sensitive parameters. Reliable bounded findings are ensured by examining the generated system’s boundedness, positivity, uniqueness, and local stability analysis, which are the crucial characteristics of epidemic models. The positive solutions with linear growth are shown to be verified by the global derivative, and the rate of impact across every sub-compartment is determined using Lipschitz criteria. Using Lyapunov functions with first derivative, the system’s global stability is examined in order to evaluate the combined effects of cytokines and anti-PD-L1 inhibitors on people with weakened immune systems. Reliability is achieved by employing the Mittag-Leffler kernel in conjunction with a fractal-fractional operator because FFO provide continuous monitoring of lung cancer in multidimensional way. The symptomatic and asymptomatic effects of lung cancer sickness are investigated using simulations in order to validate the relationship between anti-PD-L1 inhibitors, cytokines, and the immune system. Also, identify the actual state of lung cancer control with early diagnosis and therapy by introducing cytokines and anti-PD-L1 inhibitors, which aid in the patients’ production of anti-cancer cells. Investigating the transmission of illness and creating control methods based on our validated results will both benefit from this kind of research.

## Introduction

In the thirteenth century, Fibonacci proposed the famous Fibonacci sequence to describe population expansion, which marked the beginning of mathematics’ use in biology. Later, Daniel Bernoulli used mathematical ideas to explain how the impacts affected the forms of microscopic creatures, and Johannes Reinke first used the phrase “bio math” in 1901. Biomath is defined as an enzymatic research studying mathematical equations relating to the structure and the function of systems in biology. The biological sciences have had a tremendous upsurge over the last several decades, and it is realistic to anticipate that this trend will continue due to major technological breakthroughs. Mathematics has continuously benefited society and allowed it to make significant advances. Biology study may be revolutionized by mathematics, which has been instrumental in the advancement of the natural sciences^1^. Biology evaluates the mathematical models that mathematics gives in order to assist us better grasp the fascinating intricacies that biology presents. With the latest developments in computer algebra systems, solving complex mathematical problems has become simpler. Consequently, instead of attempting to solve problems, this allows the researchers to concentrate on understanding mathematical biology^2^.

Cancer is a very complicated issue that encompasses a vast range of illnesses, each with its own distinct features, totaling over 200. Consequently, a large number of researchers are still researching the relationship between tumor cells and immune cells. To learn more about the dynamics of cancer, they employ a variety of strategies^3,4^. To what extent the immune system and tumor cells interact is the purpose of this study^5^. It has been demonstrated that comprehending the dynamics of the tumor-immune system requires the application of ordinary differential equations in mathematical modeling. The development and interaction between cancer cells and host immune cells is explained by this^6–8^.

Applications of fractional calculus are well known in science, such as biology of systems^9^ and other fields^10^. You can use integrals and derivatives of non-integer order in fractional calculus. One advantage of fractional derivatives and integrals is their non-local nature^11^. Mathematical models are frequently employed in epidemiology to get a deeper comprehension of the intricacies of infectious diseases. The modeling method for dysentery with controls is analyzed using the stability theory for differential equations^12^. Local derivatives, power laws, exponential functions, and Mittag-Leffler functions are among the most often utilized operators, along with Caputo-Fabrizio and Atangana-Baleanu.

Modern cleant formalized Cancer as a monumental international issue in human health more so than AIDS, TB, and malaria all together. They note that it is prevalent in one out of every six people across the globe and is currently the second leading cause of death and prevents people from living a long life in countries that are classified by a high or very high Human Development Index (HDI)^13^. The global new cancer cases as well as an increase in the number of deaths related to cancer are increasing influenced by factors such as the improved life spans and changes in epidemiology and demography. The Sustainable Development Goal (SDG) 3.4 aims to reduce premature mortality from noncommunicable diseases (NCDs), including cancer, by 33% by 2030. Despite this, progress in cancer research and treatment has been slower compared to other NCDs. In 2018, approximately 18.1 million new cancer cases were reported, along with 9.6 million deaths due to cancer. The regional distribution of these cases was 48.4% in Asia, 21.0% in the Americas, 23.4% in Europe, 5.8% in Africa, and 1.4% in Oceania^14^.

Lung cancer stands out as the primary cause of cancer-related deaths, constituting roughly a quarter of all fatalities and surpassing the combined mortality rates of colon, breast, and prostate cancers^15^. In 2018, nations with high and medium Human Development Index (HDI) levels experienced notably higher incidences of lung cancer diagnosis in males^13^. Notably, Bangladesh recorded a prevalence of 13.1% among males and 2.0% among females over the past five years^16^. The statistics provided by the World Health Organization (WHO) for 2018 paint a concerning picture for Bangladesh, with 108,137 cancer-related deaths and 150,781 cancer diagnoses. Lung cancer contributed to 8.2% of these cases, with an associated mortality rate of 11%^17^. Furthermore, WHO projections indicate a troubling trend, with the number of lung cancer cases expected to escalate from 10,851 in 2012 to 12,374 in 2018 and a projected 26,738 instances by 2040, indicating a worsening scenario over time. By 2040, lung cancer is predicted to pose a greater threat to public health than breast cancer. There have been several researches on the subject of this paper, the dynamics of cancer. The initial mathematical model which was developed to determine the onset of cancer was by De Pillis et al.^18^. Patients also underwent injections of activation protein, TIL, and IL-2 along with their chemotherapy plan that formed the treatment regimen. Three more recent mathematical works are worth mentioning however, in this review: De Pillis et al.^19^ described a new theoretical model of tumorimmune system interaction that focused on the roles of natural killer (NK) cells and CD8+ T cells in immunologically mediating tumor rejection. In turn, Trisilowati et al.^20^ have proposed an upgraded mathematical model that considered dendritic cells for patient treatment while using natural killer cells as the core immunological element with cytotoxic T lymphocytes integrated instead of T cells. In response to these developments, Unni and Seshaiyer^21^ provided a mathematical model to address the following issues of tumor and various immune cells CD8+T cells, dendritic, and the natural killer cells. The model also spoke about the delivering of medication to the some cell location.

Kirschner and Tsygvintsev^22^ explained how their findings was used to develop a new approach of tumor treatment. The authors also provided a mathematical model to predict how the immune system responds to cancer as well. Their Hectococcus therapeutic approach relies solely on host derived factors to augment the immune response to tumors. To further explain mathematically immune-effector cells, IL-2 and tumor cells relationship, Kirschner and Panetta^23^ enhanced the mathematical model. Kartono^24^ arrived at own mathematical model that describes influence of Interleukin-2 (IL-2), interferon-alpha, and tumor-infiltrating lymphocytes on the activity of the tumor cells. Liang et al.^25^ centered their investigation on patients with small cell lung cancer receiving chemotherapy. The development of a predictive nomogram was their main goal. The precise goal of the study and the prediction algorithm that Chao and his colleagues created^26^ was to identify patients who would benefit from surgery. Furthermore, Li et al.^27^ conducted a retrospective analysis of 18 patients with complex EGFR mutations who had Non-Small Cell Lung Cancer (NSCLC), with a focus on mutations involving both common and unusual genetic abnormalities. The references^28,29^ list many forms of research done on the cancer model.

The COVID-19 model developed by the authors incorporates a harmonic mean type incidence rate, which results in a more realistic average speed^30^. Several writers investigated how population symmetry affected the development and management of a norovirus outbreak^31^. The convex incidence rate model of the Hepatitis B pandemic is studied qualitatively by the authors^32^. The authors^33^ examined the dynamic characteristics of computer virus models. The community has been severely impacted by the rabies virus propagation in recent years^34^. Quarantine and isolation compartments are added to the Mittag-Leffler kernel in this work to create the COVID-19 epidemic model^35^. The authors^36^ created a dengue epidemic model that took hospitalized class and harmonic mean incidence rate into account. The authors present a novel mathematical model intended to clarify the complex dynamics behind the spread of Anthroponotic Cutaneous Leishmania^37^. The authors created a modified immune-tumor-normal cell model, taking into account competitions of the Lotka-Volterra type between the cell populations and the chemotherapeutic medications^38^. In order to explore the impact of body mass, estrogen, and the immune system on the development of cancer cells, authors provide a mathematical model of fat, estrogen, and breast cancer^39^. After first exposing patients to chemotherapy and virotherapy alone, and then both at once, the authors examine the behaviors displayed by a modified mathematical model that depicts interactions between immune cells, uninfected tumor cells, infected tumor cells, and normal cells^40^. The goal of the authors’ work is to use the Caputo and Caputo-Fabrizio fractional operators to explore the dynamics of a nutrient-plankton system^41^. The authors^42^ investigate the function of M2 macrophages’ saturation response by addressing a tumormacrophage interaction model. The objective of the study conducted by the authors is to examine the effects of discrete-time delay on the immune response to tumor development, excess estrogen, and the source rate of immune cells in a model of breast cancer^43^.

To study the effects of different kinds of immune cells on tumor cells, many models have been developed. Nevertheless, the effect of dendritic cells on tumor cells has only been investigated in a small number of models. Furthermore, surgical techniques have not been explored as a feasible course of treatment in previous talks. On the other hand, our method incorporates both surgery and chemotherapy into our course of care. Furthermore, the authors who came before them did not clearly define the beginning of this therapeutic period. Our work, on the other hand, sets itself apart by presenting a mathematical model of non-small-cell lung cancer that includes the possible therapeutic approaches of chemotherapy and surgery and shows the combined effect of both. Additionally, we offer recommendations for the optimal approach among the various combinations involving surgery and various doses of chemotherapy.

Here, the newly introduced fractional derivatives in the analysis and numerical modelling of the lung cancer have been employed. Lung cancer is among the most dangerous types of cancer which are prevalent in human life because it has the highest tendency. This is done through establishing that the proposed system exists and has a certain set of properties, checking the validity of the solution system quantitatively. More so, by applying the Atangana Blaneao derivative, it is possible to discuss the dynamics of a mathematical model in real life. Lastly, numerical models are employed to enhance and support the biological studies conducted in the experiments. With regards to the particular importance of the aforementioned aspects, one would like to focus our analysis of these basic questions on a model developed specifically to illustrate the dynamics that are evident in lung cancer and the shortcomings of our ability to deal with it. First of all, we implemented a standard TCD setup providing a long incubation period to represent the nature of the epidemic within one population possessing a specific structure.

### Basic definition

#### Definition 1

The extended form of Riemann-Liouville fractional operator under Mittag-Leffler kernel is as follows, let $\varpi \le 1$, $0\le \zeta$. Then, *U*(*t*) is defined as follows^44^:

$$\begin{aligned} {}_{}^{FFM} D_{0,t}^{\zeta , \varpi } (U(t)) = \frac{AB(\zeta )}{1- \zeta } \frac{d}{dt^{\varpi }} \int ^{t}_{0} E_{\zeta } [- \frac{\zeta }{1- \zeta } (t-\Omega )^{\zeta } U(\Omega ) d \Omega , \end{aligned}$$

where $$0 ~ < ~ \varpi ~ \zeta , ~ \le ~ 1$$ and $$AB(\zeta ) = 1 - \zeta + \frac{\zeta }{\Gamma (\zeta )}.$$

Therefore, *U*(*t*) with a Mittag-Leffler type kernel and order $(\zeta ,\varpi )$ is defined as follows:

$$\begin{aligned} {}_{}^{FFM} D_{0,t}^{\zeta , \varpi } (U(t)) = \frac{\varpi (1-\zeta ) t^{\varpi -1} U(t)}{AB(\zeta )} + \frac{\zeta \varpi }{AB(\zeta )} \int ^{t}_{0} {\Omega }^{\zeta -1} (t-\Omega ) U(\Omega ) d \Omega . \end{aligned}$$

## Formulation of TCD$IL_{2} IL_{12}$Z model

Here, a new mathematical model is developed for lung cancer by introducing $IL_{2}$, $IL_{12}$ cytokines, and anti-PD-L1 inhibitor for treatment, whereas the TCD approach was utilized in the current model. The current model for examining cancer disease was released in June 2023 and is given in^45^. TCD$IL_{2} IL_{12}$Z is the name given to this novel model, in which “T” stands for tumor cells, “C” for CD8+ T cells, “D” for dendritic cells, “$IL_{2}$” & “$IL_{12}$” for cytokines, and “Z” for anti-PD-L1 inhibitor.

We present a number of crucial parameters in this model: The tumor’s logistic growth is described by the expression “$\alpha T (1-\beta T)$”, “$\gamma$” indicates the constant rate at which dendritic cells destroy tumor cells, “$\phi$” indicates the rate at which CD8+ T cells eliminate tumor cells, “$\kappa$” indicates the rate at which CD8+ T cells naturally die, “$\mu$” characterizes the sources that produce dendritic cells, “$\rho$” signifies the rate at which CD8+ T cells render dendritic cells inactive, “$\omega$” indicates the rate at which dendritic cells naturally die, “$\lambda$” represents the source of $IL_{2}$ to reduce the dendritic cell’s, “*d*” and “$\psi$” denoting the rate at which these cells boost the immune system through the activity of CD8+ T cells and “*a*” denoting the anti-PD-L1 inhibitor’s natural death rate.

**Figure 1:**
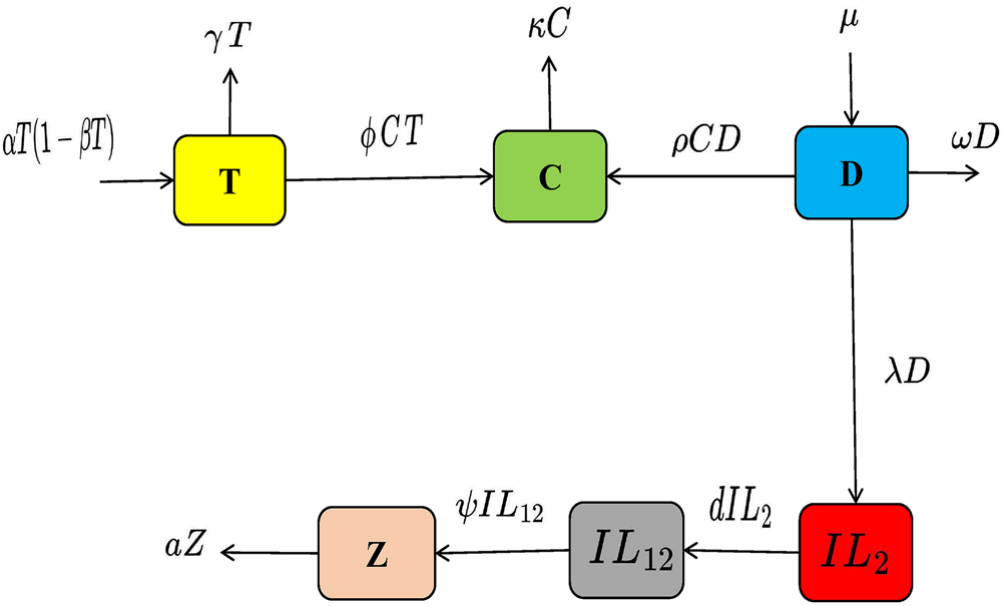
The model formulation is shown in the flow chart.

Figure 1 shows flow diagram for newly developed TCD $IL_{2} IL_{12}$Z model.

The model that was created using the anti-PD-L1 inhibitor effect and generalized hypothesis involving cytokines is shown below:

1 $$\begin{aligned} \frac{d T}{dt}= & {} \alpha T (1-\beta T)-\gamma T- \phi C T,\nonumber \\ \frac{d C}{dt}= & {} \phi C T+\rho C D-\kappa C,\nonumber \\ \frac{d D}{dt}= & {} \mu -\rho C D-\omega D-\lambda D,\nonumber \\ \frac{d IL_{2}}{dt}= & {} \lambda D-d IL_{2}, \nonumber \\ \frac{d IL_{12}}{dt}= & {} d IL_{2}-\psi IL_{12}, \nonumber \\ \frac{d Z}{dt}= & {} \psi IL_{12}-a Z. \end{aligned}$$

with initial conditions:

$$\begin{aligned} T(0)\,=\, T^{0},\, \, C(0) \,=\, C^{0}, \, \,D(0) \,=\, D^{0}, \, \,Il_{2}(0) \,=\, Il_{2}^{0},\, \, \,Il_{12}(0) \,=\, Il_{12}^{0},\, Z(0)\, =\, Z^{0}. \end{aligned}$$

When FFO is combined with Mittag-Lefller definition, the model above becomes

2 $$\begin{aligned} {}^{FFM}_{0}D^{\xi ,\lambda }_{t}T(t)= & {} \alpha T (1-\beta T)-\gamma T- \phi C T,\nonumber \\ {}^{FFM}_{0}D^{\xi ,\lambda }_{t}C(t)= & {} \phi C T+\rho C D-\kappa C,\nonumber \\ {}^{FFM}_{0}D^{\xi ,\lambda }_{t}D(t)= & {} \mu -\rho C D-\omega D-\lambda D,\nonumber \\ {}^{FFM}_{0}D^{\xi ,\lambda }_{t}IL_{2}(t)= & {} \lambda D-d IL_{2}, \nonumber \\ {}^{FFM}_{0}D^{\xi ,\lambda }_{t}IL_{12}(t)= & {} d IL_{2}-\psi IL_{12}, \nonumber \\ {}^{FFM}_{0}D^{\xi ,\lambda }_{t}Z(t)= & {} \psi IL_{12}-a Z. \end{aligned}$$

The fractal fractional operator with Mittag-lefller (FFM) in this case is $^{FFM}_{0}D^{\xi ,\lambda }_{t}$, where $0 < \xi \le 1$ and $0 < \lambda \le 1$. With initial conditions:

$$\begin{aligned} T(0)\,=\, T^{0},\, \, C(0) \,=\, C^{0}, \, \,D(0) \,=\, D^{0}, \, \,Il_{2}(0) \,=\, Il_{2}^{0},\, \, \,Il_{12}(0) \,=\, Il_{12}^{0},\, Z(0)\, =\, Z^{0}. \end{aligned}$$

## Qualitative and quantitative analysis

Within this section, I conduct a comprehensive analysis of equilibrium points. To determine these points, it is necessary to equate the left-hand side of the system (3) to 0.

$$\begin{aligned} 0= & {} \alpha T (1-\beta T)-\gamma T- \phi C T, \\ 0= & {} \phi C T+\rho C D-\kappa C,\\ 0= & {} \mu -\rho C D-\omega D-\lambda D,\\ 0= & {} \lambda D-d I L_{2},\\ 0= & {} d I L_{2}-\psi d I L_{12},\\ 0= & {} \psi d I L_{12}-a Z. \end{aligned}$$

The equilibrium point corresponding to the absence of disease in this model is

$$\begin{aligned} D_{1}(T,\,C,\,D,\,IL_{2},\,IL_{12},\,Z)=\left( \,0,\,0,\frac{\,\mu }{\omega +\lambda },\frac{\mu \lambda }{d(\omega +\lambda )},\frac{\mu \lambda }{\psi (\omega +\lambda )},\frac{\mu \lambda }{a(\omega +\lambda )}\right) . \end{aligned}$$

Furthermore, the equilibrium point associated with endemic is given as $D_{2}(T^{*},C^{*},D^{*},IL_{2}^{*},IL_{12}^{*},Z^{*})$.

where

$$\begin{aligned} T^{*}= & {} \frac{\kappa \rho \beta \alpha +\phi \rho \alpha -\phi \rho \gamma +\phi ^{2} \lambda +\omega \phi ^{2}-{\mathbb {A}}}{2\rho \beta \alpha \phi },\\ C^{*}= & {} -\frac{\kappa \rho \beta \alpha -\phi \rho \alpha +\phi \rho \gamma +\phi ^{2} \lambda +\omega \phi ^{2}-{\mathbb {A}}}{2\phi ^{2}\rho },\\ D^{*}= & {} \frac{\kappa \rho \beta \alpha -\phi \rho \alpha +\phi \rho \gamma -\phi ^{2} \lambda -\omega \phi ^{2}+{\mathbb {A}}}{2\beta \rho ^{2}\alpha },\\ IL_{2}^{*}= & {} \frac{\lambda (\kappa \rho \beta \alpha -\phi \rho \alpha +\phi \rho \gamma -\phi ^{2} \lambda -\omega \phi ^{2}+{\mathbb {A}})}{2d\beta \alpha \rho ^{2}},\\ IL_{12}^{*}= & {} \frac{\lambda (\kappa \rho \beta \alpha -\phi \rho \alpha +\phi \rho \gamma -\phi ^{2} \lambda -\omega \phi ^{2}+{\mathbb {A}})}{2\beta \alpha \rho ^{2}\psi },\\ Z^{*}= & {} \frac{\lambda (\kappa \rho \beta \alpha -\phi \rho \alpha +\phi \rho \gamma -\phi ^{2} \lambda -\omega \phi ^{2}+{\mathbb {A}})}{2a\beta \alpha \rho ^{2}}. \end{aligned}$$

where $${\mathbb {A}}=\sqrt{4\beta \alpha \mu \rho ^{2}\phi ^{2}+(\rho \alpha (\phi -\beta \kappa )+\phi (-\rho \gamma +\phi (\omega +\lambda )))^{2}}$$.

For the recently created system employing the next generation approach, the reproductive number is

$$\begin{aligned} R_{0}=\frac{\mu \rho }{\kappa (\lambda +\omega )}. \end{aligned}$$

### Sensitivity analysis

Sensitivity analysis is useful for evaluating the relative influence of several factors on the stability of a model, especially when dealing with ambiguous data. Moreover, this research helps identify the critical process factors.

Reproductive no. $``R_{0}''$ is

$$\begin{aligned} R_{0}= \frac{\mu \rho }{\kappa (\lambda +\omega )}. \end{aligned}$$

The sensitivity of $R_{0}$ can be examined by computing the partial derivatives of the threshold with respect to the pertinent parameters in the manner described below:

$$\begin{aligned} \frac{\partial R_{0}}{\partial \mu }= & {} \frac{\rho }{\kappa (\lambda +\omega )}\quad< \,0,\\ \frac{\partial R_{0}}{\partial \rho }= & {} \frac{\mu }{\kappa (\lambda +\omega )}\quad > \,0,\\ \frac{\partial R_{0}}{\partial \kappa }= & {} -\frac{\mu \rho }{\kappa ^{2}(\lambda +\omega )}\quad< \,0,\\ \frac{\partial R_{0}}{\partial \lambda }= & {} -\frac{\mu \rho }{\kappa (\lambda +\omega )^{2}} \quad< \,0,\\ \frac{\partial R_{0}}{\partial \omega }= & {} -\frac{\mu \rho }{\kappa (\lambda +\omega )^{2}} \quad < \,0, \end{aligned}$$

**Figure 2:**
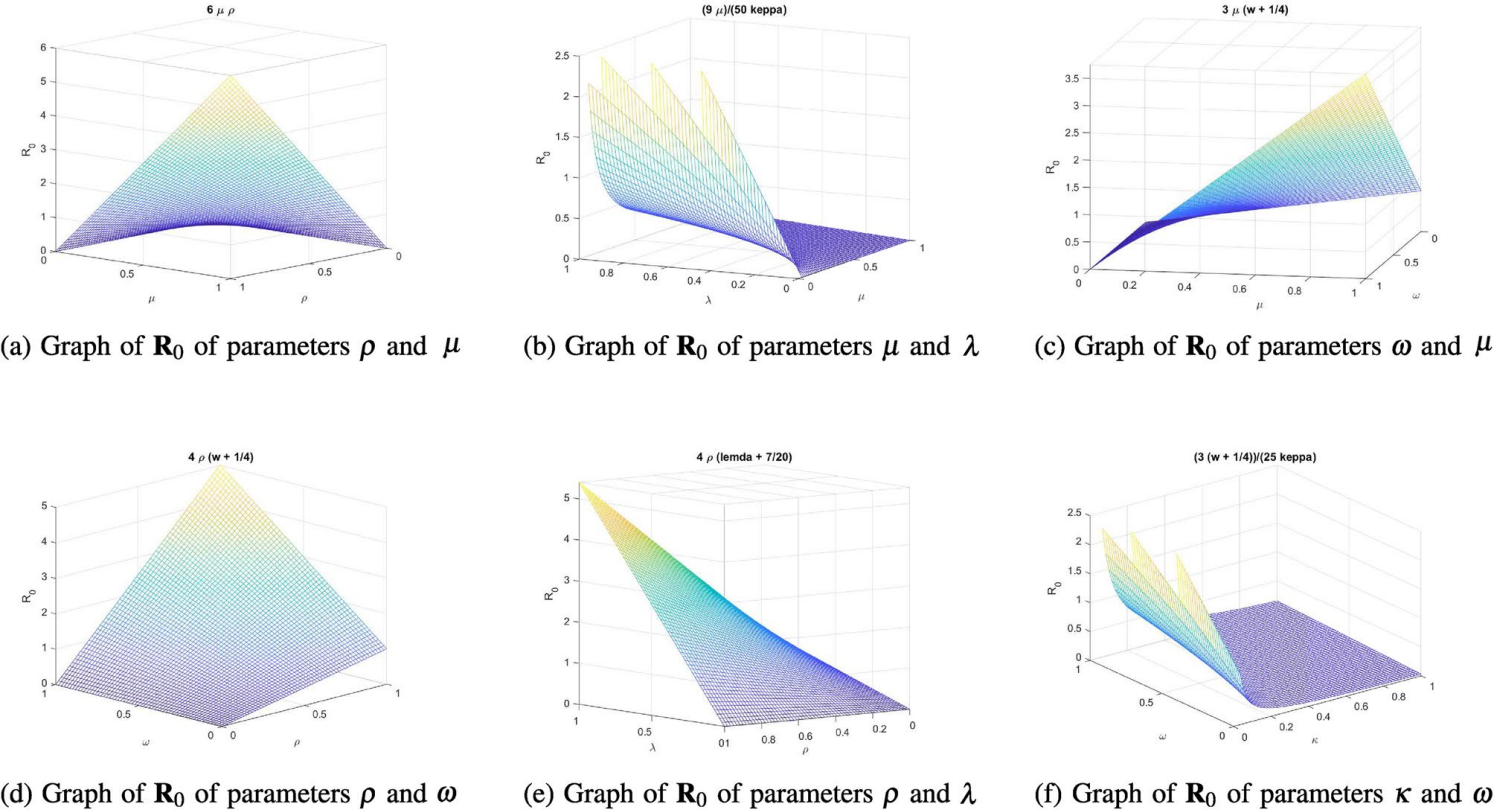
Reproductive number behavior for the newly developed system under different parameter effects.

It is evident that the value of $R_{0}$ is quite sensitive when we change the settings. We find that in our analysis, the parameters $\rho$ expand while $\mu , \kappa , \lambda , \omega$ shrink. As a result, for efficient infection management, prevention should come before therapy. As shown in Fig. 2, the previously mentioned indices aid in determining the critical elements that determine the infection’s potential for propagation.

## Local and global analysis of newly developed model

### Local stability analysis of equilibrium points

The local stability of equilibria is described here, along with related proofs.

#### Theorem 1

*The proposed fractional-order lung cancer model’s disease-free equilibrium point exhibits local asymptotic stability when* $R_0$ *is less than 1, but becomes unstable when* $R_0$ *is greater than* 1.

#### Proof

Assume the Jacobian matrix, abbreviated as ‘J’ as follows to examine the stability of the model at the points $E_0$:

$$\begin{aligned} J=\left( \begin{array}{cccccc} \alpha -2 \alpha \beta T-\gamma -\phi C &{} -\phi T &{} 0 &{} 0 &{} 0&{} 0 \\ \phi C &{} \phi T+\rho D-\kappa &{} \rho C &{} 0 &{} 0&{} 0 \\ 0 &{} -\rho D &{} -(\rho C+\omega +\lambda ) &{} 0 &{} 0 &{} 0\\ 0 &{} 0 &{} \lambda &{} -d &{} 0 &{} 0\\ 0 &{} 0 &{} 0 &{} d &{} -\psi &{} 0\\ 0 &{} 0 &{} 0 &{} d &{} \psi &{} -a \end{array} \right) \end{aligned}$$

At $$E_{0}$$

$$\begin{aligned} {J_{0}}=\left( \begin{array}{cccccc} \alpha -\gamma &{}0&{}0&{}0&{}0&{}0 \\ 0&{}\frac{\mu \rho }{\lambda +\omega }-\kappa &{}0&{}0&{}0&{}0\\ 0&{}-\frac{\mu \rho }{\lambda +\omega }&{}-\lambda -\omega &{}0&{}0&{}0\\ 0&{}0&{}\lambda &{}-d&{}0&{}0\\ 0&{}0&{}0&{}d&{}-\psi &{}0\\ 0&{}0&{}0&{}0&{}\psi &{}-a \end{array} \right) \end{aligned}$$

So,the characteristics equation is

$$\begin{aligned}{} & {} \big |J_{0}-\Lambda \,I\big |=0\\{} & {} \left| \begin{array}{cccccc} \alpha -\gamma -\Lambda &{}0&{}0&{}0&{}0&{}0 \\ 0&{}\frac{\mu \rho }{\lambda +\omega }-\kappa -\Lambda &{}0&{}0&{}0&{}0\\ 0&{}-\frac{\mu \rho }{\lambda +\omega }&{}-\lambda -\omega -\Lambda &{}0&{}0&{}0\\ 0&{}0&{}\lambda &{}-d-\Lambda &{}0&{}0\\ 0&{}0&{}0&{}d&{}-\psi -\Lambda &{}0\\ 0&{}0&{}0&{}0&{}\psi &{}-a-\Lambda \end{array}\right| =0 \end{aligned}$$

Upon solving the aforementioned determinant, we obtain the following eigenvalues $(\Lambda )$:

$$\begin{aligned} \Lambda= & {} -a,\\ \Lambda= & {} -d,\\ \Lambda= & {} \alpha -\gamma ,\\ \Lambda= & {} -\psi ,\\ \Lambda= & {} -\lambda -\omega ,\\ \Lambda= & {} \frac{-\kappa \lambda +\mu \rho -\kappa \omega }{\lambda +\omega }. \end{aligned}$$

Consequently, all of the eigenvalues have negative real parts, indicating the local asymptotic stability of the system. $\square$

### Global stability for developed system

We investigate global stability and find the criteria for illness elimination using Lyapunov’s method and LaSalle’s idea of invariance.

#### Lyapunov’s first derivative

##### Theorem 2

*If* $R_{0}>$ 1, *then the lung cancer model’s endemismic equilibrium is globally asymptotically stable*.

##### Proof

The expression for the Lyapunov function is as follows.

$$\begin{aligned} L(T^{*},C^{*},C^{*},IL_{2}^{*},Z^{*})= & {} \left( T-T^{*}-T^{*}\log \frac{T}{T^{*}}\right) +\left( C-C^{*}-C^{*}\log \frac{C}{C^{*}}\right) \\{} & {} +\left( D-D^{*}-D^{*}\log \frac{D}{D^{*}}\right) +\left( IL_{2}-IL_{2}^{*}-IL_{2}^{*}\log \frac{IL_{2}}{IL_{2}^{*}}\right) \\{} & {} +\left( IL_{12}-IL_{12}^{*}-IL_{12}^{*}\log \frac{IL_{12}}{IL_{12}^{*}}\right) +\left( Z-Z^{*}-Z^{*}\log \frac{Z}{Z^{*}}\right) . \end{aligned}$$

By using a derivative on both sides, we get

$$\begin{aligned} D_{t}^{\xi , \lambda } L= & {} \left( \frac{T-T^{*}}{T}\right) D_{t}^{\xi , \lambda } T+\left( \frac{C-C^{*}}{C}\right) D_{t}^{\xi , \lambda } C+\left( \frac{D-D^{*}}{D}\right) D_{t}^{\xi , \lambda } D\\{} & {} +\left( \frac{IL_{2}-IL_{2}^{*}}{IL_{2}}\right) D_{t}^{\xi , \lambda } IL_{2}+\left( \frac{IL_{12}-IL_{12}^{*}}{IL_{12}}\right) D_{t}^{\xi , \lambda }IL_{12}\\{} & {} +\left( \frac{Z-Z^{*}}{Z}\right) D_{t}^{\xi , \lambda } Z. \end{aligned}$$

We get,

$$\begin{aligned} D_{t}^{\xi , \lambda } L= & {} \left( \frac{T-T^{*}}{T}\right) \left( \alpha T (1-\beta T)-\gamma T- \phi C T\right) \\{} & {} +\left( \frac{C-C^{*}}{C}\right) \left( \phi C T+\rho C D-\kappa C\right) \\{} & {} +\left( \frac{D-D^{*}}{D}\right) \left( \mu -\rho C D-\omega D-\lambda D\right) \\{} & {} +\left( \frac{IL_{2}-IL_{2}^{*}}{IL_{2}}\right) \left( \lambda D-d IL_{2}\right) \\{} & {} +\left( \frac{IL_{12}-IL_{12}^{*}}{IL_{12}}\right) \left( d IL_{2}-\psi IL_{12} \right) +\left( \frac{Z-Z^{*}}{Z}\right) \left( \psi IL_{12}-a Z\right) . \end{aligned}$$

placing $T=T-T^{*},C=C-C^{*},D=D-D^{*},IL_{2}=IL_{2}-IL_{2}^{*},IL_{12}=IL_{12}-IL_{12}^{*},Z=Z-Z^{*}$ results in

$$\begin{aligned} D_{t}^{\xi , \lambda } L= & {} \alpha \frac{(T-T^{*})^{2}}{T}-\alpha \beta \frac{(T-T^{*})^{3}}{T}-\gamma \frac{(T-T^{*})^{2}}{T}-\phi C \frac{(T-T^{*})^{2}}{T}\\{} & {} +\phi C^{*} \frac{(T-T^{*})^{2}}{T}+\phi T \frac{(C-C^{*})^{2}}{C}-\phi T^{*} \frac{(C-C^{*})^{2}}{C}\\{} & {} + \rho D \frac{(C-C^{*})^{2}}{C}-\rho D^{*} \frac{(C-C^{*})^{2}}{C}-\kappa \frac{(C-C^{*})^{2}}{C}+\mu -\mu \frac{D^{*}}{D}\\{} & {} -\rho C \frac{(D-D^{*})^{2}}{D}+\rho C^{*} \frac{(D-D^{*})^{2}}{D}-\omega \frac{(D-D^{*})^{2}}{D}\\{} & {} - \lambda \frac{(D-D^{*})^{2}}{D}+ \lambda D-\lambda D \frac{IL_{2}^{*}}{IL_{2}}-\lambda D^{*}+\lambda D^{*} \frac{IL_{2}^{*}}{IL_{2}}\\{} & {} -d \frac{(IL_{2}-IL_{2}^{*})^{2}}{IL_{2}}+d IL_{2}-d IL_{2}^{*}-d IL_{2} \frac{IL_{12}^{*}}{IL_{12}}+d IL_{2}^{*} \frac{IL_{12}^{*}}{IL_{12}}\\{} & {} -\psi \frac{(IL_{12}-IL_{12}^{*})^{2}}{IL_{12}}+\psi IL_{12}-\psi IL_{12}^{*} \\{} & {} -\psi IL_{12} \frac{Z^{*}}{Z}+\psi IL_{12}^{*} \frac{Z^{*}}{Z}- a \frac{(Z-Z^{*})^{2}}{Z}. \end{aligned}$$

we can write

$$\begin{aligned} D_{t}^{\xi , \lambda } L =\Sigma -\Omega . \end{aligned}$$

where

$$\begin{aligned} \Sigma= & {} \alpha \frac{(T-T^{*})^{2}}{T}+\phi C^{*} \frac{(T-T^{*})^{2}}{T}+\phi T \frac{(C-C^{*})^{2}}{C}+\rho D \frac{(C-C^{*})^{2}}{C}\\{} & {} +\mu +\rho C^{*} \frac{(D-D^{*})^{2}}{D} + \lambda D+\lambda D^{*} \frac{IL_{2}^{*}}{IL_{2}}+d IL_{2}+d IL_{2}^{*} \frac{IL_{12}^{*}}{IL_{12}}\\{} & {} +\psi IL_{12}-\psi IL_{12}^{*} +\psi IL_{12}^{*} \frac{Z^{*}}{Z}. \end{aligned}$$

and

$$\begin{aligned} \Omega= & {} \alpha \beta \frac{(T-T^{*})^{3}}{T}+\gamma \frac{(T-T^{*})^{2}}{T}+\phi C \frac{(T-T^{*})^{2}}{T}+\phi T^{*} \frac{(C-C^{*})^{2}}{C}\\{} & {} +\rho D^{*} \frac{(C-C^{*})^{2}}{C}+\kappa \frac{(C-C^{*})^{2}}{C}+\mu \frac{D^{*}}{D}+\rho C \frac{(D-D^{*})^{2}}{D}\\{} & {} +\omega \frac{(D-D^{*})^{2}}{D}+\lambda \frac{(D-D^{*})^{2}}{D}+ \lambda D \frac{IL_{2}^{*}}{IL_{2}}+\lambda D^{*}+d \frac{(IL_{2}-IL_{2}^{*})^{2}}{IL_{2}}+d IL_{2}^{*}+d IL_{2} \frac{IL_{12}^{*}}{IL_{12}}\\{} & {} +\psi \frac{(IL_{12}-IL_{12}^{*})^{2}}{IL_{12}}+\psi IL_{12} \frac{Z^{*}}{Z}+ a \frac{(Z-Z^{*})^{2}}{Z}. \end{aligned}$$

It can be concluded that when $\Sigma <\Omega$, $D_{t}^{\xi , \lambda } L <0$ is obtained; on the other hand, when $T=T^{*}, C=C^{*}, D=D^{*}, IL_{2}=IL_{12}^{*}, IL_{12}=IL_{12}^{*}, Z=Z^{*}$.

$$\begin{aligned} \Sigma -\Omega =0 \hspace{0.15cm} \Rightarrow \quad D_{t}^{\xi , \lambda } L =0. \end{aligned}$$

As we can see,

$\{(T^{*}, C^{*}, D^{*}, IL_{12}^{*}, IL_{12}^{*}, Z^{*})\in \Gamma : \quad D_{t}^{\xi , \lambda } L =0\}$ denotes the created model’s point $D_2$.

If $\Sigma <\Omega$, then the $D_2$ is globally uniformly stable in $\Gamma$, in accordance with Lasalles’ idea of invariance. $\square$

### Chaos control

System (3) is a fractional-order system with a controlled design that is stabilized based on its points of equilibrium through the use of the linear feedback regulate technique.

$$\begin{aligned} \begin{aligned} { }_0^{F F M} D_t^{\xi , \lambda } T(t)&=\alpha T(1-\beta T)-\gamma T-\phi C T -\omega _1(T-T^*), \\ { }_0^{F F M} D_t^{\xi , \lambda } C(t)&=\phi C T+\rho C D-\kappa C-\omega _2(C-C^*), \\ { }_0^{F F M} D_t^{\xi , \lambda } D(t)&=\mu -\rho C D-\omega D-\lambda D-\omega _3(D-D^*), \\ { }_0^{F F M} D_t^{\xi , \lambda } I L_2(t)&=\lambda D-d I L_2-\omega _4(IL_2-IL_2^*), \\ { }_0^{F F M} D_t^{\xi , \lambda } I L_{12}(t)&=d I L_2-\psi I L_{12}-\omega _5(IL_{12}-IL{12}^*), \\ { }_0^{F F M} D_t^{\xi , \lambda } Z(t)&=\psi I L_{12}-a Z-\omega _6(Z-Z^*). \end{aligned} \end{aligned}$$

where the system’s equilibrium is represented by $D_1$ and the control parameters are $\omega _1$, $\omega _2$, $\omega _3$, $\omega _4$, $\omega _5$, and $\omega _6$. At $D_1$, the related Jacobian matrix is found as follows: At $D_1$

$$\begin{aligned} J_0=\left( \begin{array}{cccccc} \alpha -\gamma -\omega _1 &{} 0 &{} 0 &{} 0 &{} 0 &{} 0 \\ 0 &{} \frac{\mu \rho }{\lambda +\omega }-\kappa -\omega _2 &{} 0 &{} 0 &{} 0 &{} 0 \\ 0 &{} -\frac{\mu \rho }{\lambda +\omega } &{} -\lambda -\omega -\omega _3 &{} 0 &{} 0 &{} 0 \\ 0 &{} 0 &{} \lambda &{} -d-\omega _4 &{} 0 &{} 0 \\ 0 &{} 0 &{} 0 &{} d &{} -\psi -\omega _5 &{} 0 \\ 0 &{} 0 &{} 0 &{} 0 &{} \psi &{} -a-\omega _6 \end{array}\right) \end{aligned}$$

Thus, the characteristics equation is

$$\begin{aligned}{} & {} \left| J_0-\Lambda I\right| =0\\{} & {} \left| \begin{array}{cccccc} \alpha -\gamma -\Lambda -\omega _1 &{} 0 &{} 0 &{} 0 &{} 0 &{} 0 \\ 0 &{} \frac{\mu \rho }{\lambda +\omega }-\kappa -\Lambda -\omega _2 &{} 0 &{} 0 &{} 0 &{} 0 \\ 0 &{} -\frac{\mu \rho }{\lambda +\omega } &{} -\lambda -\omega -\Lambda -\omega _3 &{} 0 &{} 0 &{} 0 \\ 0 &{} 0 &{} \lambda &{} -d-\Lambda -\omega _4 &{} 0 &{} 0 \\ 0 &{} 0 &{} 0 &{} d &{} -\psi -\Lambda -\omega _5 &{} 0 \\ 0 &{} 0 &{} 0 &{} 0 &{} \psi &{} -a-\Lambda -\omega _6 \end{array}\right| =0 \end{aligned}$$

The following are the eigenvalues $(\Lambda )$ that result from solving the aforementioned determinant:

$$\begin{aligned} \begin{aligned} \Lambda&=-1.0486 \\ \Lambda&=-0.5238 \\ \Lambda&=-3.2400 \\ \Lambda&=-4.003 \\ \Lambda&=-5.003 \\ \Lambda&=-6.0400. \end{aligned} \end{aligned}$$

Consequently, all of the eigenvalues have negative real parts, indicating the local asymptotic stability of the system. At $D_2$

$$\begin{aligned} J_0=\left( \begin{array}{cccccc} \alpha -\gamma -\omega _1-\phi C^*-2\alpha \beta T^* &{} -\phi T &{} 0 &{} 0 &{} 0 &{} 0 \\ \phi C^* &{} \phi T^*+\rho D^*-\kappa -\omega _2 &{} \rho C^* &{} 0 &{} 0 &{} 0 \\ 0 &{} -\frac{\mu \rho }{\lambda +\omega } &{} -\rho C^* -\lambda -\omega -\omega _3 &{} 0 &{} 0 &{} 0 \\ 0 &{} -\rho D^* &{} \lambda &{} -d-\omega _4 &{} 0 &{} 0 \\ 0 &{} 0 &{} 0 &{} d &{} -\psi -\omega _5 &{} 0 \\ 0 &{} 0 &{} 0 &{} d &{} \psi &{} -a-\omega _6 \end{array}\right) \end{aligned}$$

Thus, the characteristics equation is

$$\begin{aligned}{} & {} \left| J_0-\Lambda I\right| =0\\{} & {} \left| \begin{array}{cccccc} \alpha -\gamma -\Lambda -\omega _1-\phi C^*-2\alpha \beta T^* &{} -\phi T &{} 0 &{} 0 &{} 0 &{} 0 \\ \phi C^* &{} \phi T^*+\rho D^*-\kappa -\Lambda -\omega _2 &{} \rho C^* &{} 0 &{} 0 &{} 0 \\ 0 &{} -\frac{\mu \rho }{\lambda +\omega } &{}-\rho C^* -\lambda -\omega -\Lambda -\omega _3 &{} 0 &{} 0 &{} 0 \\ 0 &{} -\rho D^* &{} \lambda &{} -d-\Lambda -\omega _4 &{} 0 &{} 0 \\ 0 &{} 0 &{} 0 &{} d &{} -\psi -\Lambda -\omega _5 &{} 0 \\ 0 &{} 0 &{} 0 &{} d &{} \psi &{} -a-\Lambda -\omega _6 \end{array}\right| =0 \end{aligned}$$

The following are the eigenvalues $(\Lambda )$ that result from solving the aforementioned determinant:

$$\begin{aligned} \begin{aligned} \Lambda&=-1.0486 \\ \Lambda&=-2.0200 \\ \Lambda&=-3.2400 \\ \Lambda&=-4.003 \\ \Lambda&=-5.003 \\ \Lambda&=-6.0400. \end{aligned} \end{aligned}$$

As a result, all of the eigenvalues bear negative real parts and this proves that it is locally asymptotically stable. In each of the above equated is eigenvalue, negative real number or a complex number with negative real parts therefore, for any $$0<q<5$$, equilibrium points $$ D_1,$$ $$D_2$$ are asymptotic stability.

## Bounded and positive solutions

We illustrate the derived model’s positivity and boundedness in this section.

### Theorem 3

*The initial values under consideration as*:

$$\begin{aligned} \{T^{0},C^{0}, D^{0},IL_{2}^{0},IL_{12},Z^{0}\}\subset \Upsilon , \end{aligned}$$

*Consequently*, $\{ T,C,D,IL_{2},IL_{12},Z \}$ *will have positive solutions, with* $\forall$ t $\ge$.

### Proof

To demonstrate the higher caliber of the answers, we shall start the main analysis. These methods have good effects and successfully handle problems in the actual world. The methods described in references^46–48^ will be employed by us. We’ll look at the prerequisites in this section to make sure the recently created model produces results that are favorable. We shall set the standard in order to achieve this:

$$\begin{aligned} \parallel \eta \parallel _{\infty }=\sup _{t\in D_{\eta }}\mid \eta (t)\mid , \end{aligned}$$

here “$D_{\eta }$” represents the $\eta$ domain. Now, for *T*(*t*):

$$\begin{aligned} {}^{FFM}_{0}D^{\xi ,\lambda }_{t}T(t)= & {} \alpha T (1-\beta T)-\gamma T- \phi C T,\hspace{0.5cm}\forall t \ge 0,\\\ge & {} -\left( \gamma + \phi \parallel C \parallel _{\infty }+ \beta \alpha \parallel T \parallel _{\infty } \right) T,\hspace{0.5cm}\forall t \ge 0. \end{aligned}$$

we get,

$$\begin{aligned} T(t)\ge T(0)E_{\xi }\bigg [-\frac{b^{1-\lambda }{\xi }{(\alpha \beta \parallel T \parallel _{\infty }+\gamma +\phi \parallel C \parallel _{\infty })}{t^{\xi }}}{{A B}(\xi )-(1-\xi )(\alpha \beta \parallel T \parallel _{\infty }+\gamma +\phi \parallel C \parallel _{\infty })}\bigg ],\hspace{0.5cm}\forall t \ge 0, \end{aligned}$$

here the time component is denoted by $``b''$. This proves that $\forall \,\,t \ge$ 0, *T*(*t*) must be positive. Now, for *C*(*t*):

$$\begin{aligned} {}^{FFM}_{0}D^{\xi ,\lambda }_{t}C(t)= & {} \phi C T+\rho C D-\kappa C,\hspace{0.5cm}\forall \,\, t \ge 0,\\\ge & {} -(-\rho \parallel D \parallel _{\infty }+\kappa -\phi \parallel T \parallel _{\infty }) C,\hspace{0.5cm}\forall \,\,t \ge 0. \end{aligned}$$

we get,

$$\begin{aligned} C(t)\ge C(0)E_{\xi }\bigg [-\frac{b^{1-\lambda }{\xi }{(-\rho \parallel D \parallel _{\infty }+\kappa -\phi \parallel T \parallel _{\infty })}{t^{\xi }}}{A B(\xi )-(1-\xi )(-\rho \parallel D \parallel _{\infty }+\kappa -\phi \parallel T \parallel _{\infty })}\bigg ],\hspace{0.5cm}\forall \,\,t \ge 0, \end{aligned}$$

here the time component is denoted by $``b''$. This proves that $\forall \,\,t \ge$ 0, *C*(*t*) must be positive. Now, for *D*(*t*):

$$\begin{aligned} {}^{FFM}_{0}D^{\xi ,\lambda }_{t}D(t)= & {} \mu -\rho C D-(\omega + \lambda ) D,\hspace{0.5cm}\forall t \ge 0,\\\ge & {} -(\lambda +\rho \parallel C \parallel _{\infty }+\omega )D,\hspace{0.5cm}\forall t \ge 0. \end{aligned}$$

we get,

$$\begin{aligned} D(t)\ge D(0)E_{\xi }\bigg [-\frac{b^{1-\lambda {\xi }}{(\lambda +\omega +\rho \parallel C \parallel _{\infty })}{t^{\xi }}}{A B(\xi )-(1-\xi )\left( \lambda +\omega +\rho \parallel C \parallel _{\infty }\right) }\bigg ],\hspace{0.5cm}\forall t \ge 0, \end{aligned}$$

here the time component is denoted by $``b''$. This proves that $\forall \,\,t \ge$ 0, *D*(*t*) must be positive. Now, for $IL_{2}(t)$:

$$\begin{aligned} {}^{FFM}_{0}D^{\xi ,\lambda }_{t}IL_{2}(t)= & {} \lambda D-d IL_{2},\hspace{0.5cm}\forall t \ge 0,\\\ge & {} - d IL_{2},\hspace{0.5cm}\forall t \ge 0. \end{aligned}$$

we get,

$$\begin{aligned} IL_{2}(t)\ge IL_{2}(0)E_{\xi }\bigg [-\frac{b^{1-\lambda }{\xi }{(d)}{t^{\xi }}}{A B(\,\xi )\,-\,(1\,-\,\xi )(d)}\bigg ],\hspace{0.5cm}\forall t \ge 0, \end{aligned}$$

here the time component is denoted by $``b''$. This proves that $\forall \,\,t \ge$ 0, *T*(*t*) must be positive. Now, for $IL_{12}(t)$:

$$\begin{aligned} {}^{FFM}_{0}D^{\xi ,\lambda }_{t}IL_{12}(t)= & {} d IL_{2}-\psi IL_{12},\hspace{0.5cm}\forall t\ge 0,\\\ge & {} -\psi IL_{12},\hspace{0.5cm}\forall t\ge 0. \end{aligned}$$

we get,

$$\begin{aligned} IL_{12}(t)\ge IL_{12}(0)E_{\xi }\bigg [-\frac{b^{1-\lambda }{\xi }{(\psi )}{t^{\xi }}}{A B(\xi )-(1-\xi )(\psi )}\bigg ],\hspace{0.5cm}\forall t\ge 0, \end{aligned}$$

here the time component is denoted by $``b''$. This proves that $\forall \,\,t \ge$ 0, *T*(*t*) must be positive. Now, for *Z*(*t*):

$$\begin{aligned} {}^{FFM}_{0}D^{\xi ,\lambda }_{t}Z(t)= & {} \psi IL_{12}-a Z,\hspace{0.5cm}\forall \,t\,\ge \,0,\\\ge & {} -a Z(t),\hspace{0.5cm}\forall \,t\,\ge \,0. \end{aligned}$$

we get,

$$\begin{aligned} Z(t)\ge Z(0){E_{\xi }}\bigg [-\frac{b^{1-\lambda }\xi {(a)}{t^{\xi }}}{{A B}(\xi )-(1-\xi )(a)}\bigg ],\hspace{0.5cm}\forall t\ge 0, \end{aligned}$$

here the time component is denoted by $``b''$. This proves that $\forall \,\,t \ge$ 0, *T*(*t*) must be positive. $\square$

### Theorem 4

*With positive initial values, all results of developed model, as expressed in Eq.* (3), *are bounded*.

### Proof

As can be seen from the above theorem, methods are detailed in^49^. The answers of our constructed model must be positive $\forall \,\,t\,\ge \,0$. Given that $X = T\,+\,C\,+\,D$. so provided as follows.

$$\begin{aligned} {}^{FFM}_{0} D^{\xi ,\lambda }_{t} X(t)=\delta X+{\textbf {a}}-(\mu _{0}+\phi -{\textbf {b}}+\omega )I. \end{aligned}$$

We get,

$$\begin{aligned} \Psi _{p}=\{T,\,C,\,D \in R_{+}^{3} \mid D + T \le X\}\hspace{0.4cm}\forall \,\,t \ge 0. \end{aligned}$$

Additionally, it has $X_{\upsilon }=IL_{2}+IL_{12}+Z$. We have so evolved.

$$\begin{aligned} {}^{FFM}_{0}D^{\xi ,\lambda }_{t}X_{\upsilon }(t)= \lambda D-a Z+X_{\upsilon }-X_{\upsilon }, \end{aligned}$$

After calculating $t\rightarrow \infty$ and solving the preceding equation, we obtain

$$\begin{aligned} X_{\upsilon }\le \lambda \,D-a Z+X_{\upsilon }. \end{aligned}$$

Thus

$$\begin{aligned} \Psi _{\upsilon }=\bigg \{IL_{2},\,IL_{12},\,\,Z\in R_{+}^{3}\mid X_{\upsilon }\le \lambda D-a Z+X_{\upsilon }\bigg \}\hspace{0.4cm}\forall \,\,t \ge 0. \end{aligned}$$

The mathematical solutions (3) for the model are limited to $\Psi$.

$$\begin{aligned} \Psi =\bigg \{T,\,C,\,D,\,\,IL_{2},\,IL_{12},\,Z \in R_{+}^{6}\mid D + T\le X, X_{\upsilon } \le \lambda \,D-a Z+X_{\upsilon }\bigg \}\hspace{0.4cm}\forall \,\,t \ge 0. \end{aligned}$$

This proves that all solutions in domain $\Psi$ stay positive and consistent with given beginning conditions for any $t \ge$ 0. $\square$

### Theorem 5

*In addition to the beginning condition, the recently created lung cancer model* Eq. (3) *in* $R_{+}^{6}$ *is unique and positive invariant*.

### Proof

In this specific case, we used the process outlined in^49^. We possess

3 $$\begin{aligned} {}^{FFM}_{0}D^{\xi ,\lambda }_{t}(T(t))_{T=0}= & {} \alpha T \hspace{0.5cm}\ge 0,\nonumber \\ {}^{FFM}_{0}D^{\xi ,\lambda }_{t}(C(t))_{C=0}= & {} \phi C T+\rho C D \hspace{0.5cm}\ge 0,\nonumber \\ {}^{FFM}_{0}D^{\xi ,\lambda }_{t}(D(t))_{D=0}= & {} \mu \hspace{0.5cm}\ge 0,\nonumber \\ {}^{FFM}_{0}D^{\xi ,\lambda }_{t}(IL_{2}(t))_{IL_{2}=0}= & {} \lambda D\hspace{0.5cm}\ge 0,\nonumber \\ {}^{FFM}_{0}D^{\xi ,\lambda }_{t}(IL_{2}(t))_{IL_{12}=0}= & {} d IL_{2}\hspace{0.5cm}\ge 0,\nonumber \\ {}^{FFM}_{0}D^{\xi ,\lambda }_{t}(Z(t))_{Z=0}= & {} \psi IL_{12} \hspace{0.5cm}\ge 0. \end{aligned}$$

Equation (4) states that if $(T^{0}, C^{0}, D^{0}, IL_{2}^{0}, IL_{12}^{0}, Z^{0})$ $\in$ $R_{+}^{6}$, then our obtained solution cannot escape from the hyperplane. This demonstrates that the domain $R_{+}^{6}$ becomes a positive invariant. $\square$

## Impact of global derivatives for existence and uniqueness of solution

The most often used integral in the literature is generally acknowledged to be the Riemann–Stieltjes integral. If

$$\begin{aligned} Y(x)=\int y(x){d}x. \end{aligned}$$

Riemann-Stieltjes integral is:

$$\begin{aligned} Y_{n}(x)=\int y(x){d}{n}(x), \end{aligned}$$

The global derivative of *y*(*x*) with respect to *n*(*x*) is

$$\begin{aligned} D_{n}y(x)=\lim _{h\rightarrow 0}\frac{y(x+h)-y(x)}{n(x+h)-n(x)}. \end{aligned}$$

When the numerator and denominator of the aforementioned functions differentiate, we obtain

$$\begin{aligned} D_{n}y(x)=\frac{y^{'}(x)}{n^{'}(x)}, \end{aligned}$$

Considering the following: $n^{'}(x)\,\,\ne$ 0, $\forall x\in D_{n^{'}}$. Using the global derivative in place of the conventional derivative, we will now examine the effect on the lung cancer.

$$\begin{aligned} D_{n}T= & {} \alpha T (1-\beta T)-\gamma T- \phi C T,\\ D_{n}C= & {} \phi C T+\rho C D-\kappa C,\\ D_{n}D= & {} \mu -\rho C D-\omega D-\lambda D,\\ D_{n}IL_{2}= & {} \lambda D-d IL_{2},\\ D_{n}IL_{12}= & {} d IL_{2}-\psi IL_{12},\\ D_{n}Z= & {} \psi IL_{12}-a Z. \end{aligned}$$

We shall assume that n is differentiable for the sake of cleanliness.

$$\begin{aligned} T^{'}= & {} n^{'}[\alpha T (1-\beta T)-\gamma T- \phi C T],\\ C^{'}= & {} n^{'}[\phi C T+\rho C D-\kappa C],\\ D^{'}= & {} n^{'}[\mu -\rho C D-\omega D-\lambda D],\\ IL_{2}^{'}= & {} n^{'}[\lambda D-d IL_{2}],\\ IL_{12}^{'}= & {} n^{'}[d IL_{2}-\psi IL_{12}],\\ Z^{'}= & {} n^{'}[\psi IL_{12}-a Z]. \end{aligned}$$

An proper selection of the function *n*(*t*) will result in a certain result. Fractal movement will be seen, for example, if $n(t)=t^{\eta }$, where $\eta$ is a real value. The conditions that required us to act were

$$\begin{aligned} \parallel n^{'}\parallel _{\infty }=\sup _{t\in D_{n^{'}}}\mid n^{'}(t)\mid <N. \end{aligned}$$

The system’s unique solution is shown in the example below for the created system.

$$\begin{aligned} T^{'}= & {} n^{'}[\alpha T (1-\beta T)-\gamma T- \phi C T]=X_{1}(t, T, H),\\ C^{'}= & {} n^{'}[\phi C T+\rho C D-\kappa C]=X_{2}(t, T, H),\\ D^{'}= & {} n^{'}[\mu -\rho C D-\omega D-\lambda D]=X_{3}(t, T, H), \\ IL_{2}^{'}= & {} n^{'}[\lambda D-d IL_{2}]=X_{4}(t, T, H),\\ IL_{12}^{'}= & {} n^{'}[d IL_{2}-\psi IL_{12}]=X_{5}(t, T, H),\\ Z^{'}= & {} n^{'}[\psi IL_{12}-a Z]=X_{6}(t, T, H). \end{aligned}$$

where $$H = C,D,IL_{2},IL_{12},Z$$.

The first two conditions are as follows, which we must verify.

1.$$\, \, \mid X(\,t, \,T, \,H)\,\mid ^{2} < K(\,1\,+\,\mid T \mid ^{2}$$, 2.$$\, \, \hspace{0.1cm} \forall \hspace{0.1cm}T_{1},T_{2}$$, we  have,

$$\parallel X(t,T_{1}, H)-X(t,T_{2}, H)\parallel ^{2} < \bar{K}\parallel T_{1}-T_{2}\parallel _{\infty }^{2}$$.

Initially,

$$\begin{aligned} \mid X_{1}(t, T, H)\mid ^{2}= & {} \mid n^{'}\big [\alpha T (1-\beta T)-\gamma T- \phi C T\big ]\mid ^{2},\\\le & {} 2\sup _{t\in D_{n^{'}}}\mid n^{'}\mid ^{2}\alpha ^{2}\sup _{t\in D_{T}}\mid T \mid ^{2}+6\sup _{t\in D_{n^{'}}}\mid n^{'}\mid ^{2}\left( \alpha ^{2} \beta ^{2}\sup _{t\in D_{T}}\mid T \mid ^{2}+\gamma ^{2}+\phi ^{2} \sup _{t\in D_{C}}\mid C \mid ^{2}\right) \mid T \mid ^{2},\\\le & {} 2 \parallel n^{'}\parallel ^{2}_{\infty } \parallel T \parallel ^{2}_{\infty } \alpha ^{2}\left( 1+\frac{3}{\parallel T \parallel ^{2}_{\infty } \alpha ^{2}}\left( \beta ^{2} \alpha ^{2} \parallel T \parallel ^{2}_{\infty }+ \phi ^{2} \parallel C \parallel ^{2}_{\infty } + \gamma ^{2}\right) \mid T \mid ^{2}\right) ,\\\le & {} K_{1}(1\,+\mid \,T \,\mid ^{2}). \end{aligned}$$

for condition:

$$\begin{aligned} \frac{3}{\parallel T \parallel ^{2}_{\infty } \alpha ^{2}}\left( \beta ^{2} \alpha ^{2} \parallel T \parallel ^{2}_{\infty }+ \phi ^{2} \parallel C \parallel ^{2}_{\infty } + \gamma ^{2}\right) \,<\,1, \end{aligned}$$

where

$$\begin{aligned} K_{1}= & {} 2\parallel n^{'}\parallel ^{2}_{\infty } \parallel T \parallel ^{2}_{\infty } \alpha ^{2}.\\ \mid X_{2}(t, T, H)\mid ^{2}= & {} \mid n^{'}\big [\phi C T+\rho C D-\kappa C\big ]\mid ^{2},\\\le & {} 2\parallel n^{'}\parallel ^{2}_{\infty }\kappa ^{2}\parallel C \parallel ^{2}_{\infty }+4\parallel n^{'}\parallel ^{2}_{\infty }(\parallel T \parallel ^{2}_{\infty } \phi ^{2} + \parallel D \parallel ^{2}_{\infty } \rho ^{2})\mid C \mid ^{2},\\\le & {} K_{2}(1+\mid C \mid ^{2}). \end{aligned}$$

for condition:

$$\begin{aligned} \frac{2(\parallel T \parallel ^{2}_{\infty } \phi ^{2} + \parallel D \parallel ^{2}_{\infty } \rho ^{2})}{\kappa ^{2}\parallel C \parallel ^{2}_{\infty }}<1, \end{aligned}$$

where

$$\begin{aligned} K_{2}=2\parallel n^{'}\parallel ^{2}_{\infty }\kappa ^{2}\parallel C \parallel ^{2}_{\infty }.\\ \mid X_{3}(t, T, H)\mid ^{2}= & {} \mid n^{'}\big [\,\mu -\rho C D-(\lambda +\omega ) D \big ]\mid ^{2},\\\le & {} 2 \mu ^{2} \parallel n^{'}\parallel ^{2}_{\infty }\left( 1+\frac{3}{\mu ^{2}}\left( \omega ^{2} + \parallel C \parallel ^{2}_{\infty } \rho ^{2} +\lambda ^{2}\right) \mid D \mid ^{2}\right) ,\\\le & {} K_{3}(1+\mid D \mid ^{2}). \end{aligned}$$

for condition:

$$\begin{aligned} \frac{3}{\mu ^{2}}\left( \omega ^{2} + \rho ^{2} \parallel C \parallel ^{2}_{\infty }+\lambda ^{2}\right) <1, \end{aligned}$$

where

$$\begin{aligned} K_{3}= & {} 2 \parallel \mid n^{'}\parallel ^{2}_{\infty } \mu ^{2}.\\ \mid X_{4}(t, T, H)\mid ^{2}= & {} \mid n^{'}\big [\,D-d IL_{2+\lambda }\big ]\mid ^{2},\\\le & {} 2\parallel n^{'}\parallel ^{2}_{\infty } \parallel D \parallel ^{2}_{\infty } \lambda ^{2} \left( 1+\frac{d^{2}\mid IL_{2}\mid ^{2}}{\lambda ^{2}\parallel D \parallel ^{2}_{\infty }}\right) ,\\\le & {} K_{4}(1+\mid IL_{2}\mid ^{2}). \end{aligned}$$

for condition:

$$\begin{aligned} \frac{d^{2}}{\lambda ^{2}\parallel D \parallel ^{2}_{\infty }}<1, \end{aligned}$$

where

$$\begin{aligned} K_{4}= & {} 2\parallel n^{'}\parallel ^{2}_{\infty } \parallel D \parallel ^{2}_{\infty } \lambda ^{2}.\\ \mid X_{5}(t, T, H)\mid ^{2}= & {} \mid n^{'}\big [d IL_{2}-\psi IL_{12}\big ]\mid ^{2},\\\le & {} 2 d^{2} \parallel n^{'}\parallel ^{2}_{\infty }\parallel IL_{2} \parallel ^{2}_{\infty }\left( 1+\frac{\psi ^{2}\mid IL_{12}\mid ^{2}}{d^{2}\parallel IL_{2} \parallel ^{2}_{\infty }}\right) ,\\\le & {} K_{5}(1+\mid IL_{12}\mid ^{2}). \end{aligned}$$

for condition:

$$\begin{aligned} \frac{\psi ^{2}}{d^{2}\parallel IL_{2} \parallel ^{2}_{\infty }}<1, \end{aligned}$$

where

$$\begin{aligned} K_{5}= & {} 2\parallel n^{'}\parallel ^{2}_{\infty }d^{2}\parallel IL_{2} \parallel ^{2}_{\infty }.\\ \mid X_{6}(t, T, H)\mid ^{2}= & {} \mid n^{'}\big [\psi IL_{12}-a Z\big ]\mid ^{2},\\\le & {} 2 \parallel n^{'}\parallel ^{2}_{\infty }\left( \parallel IL_{12} \parallel ^{2}_{\infty } \psi ^{2}\right) \left( 1+\frac{a^{2}\mid Z \mid ^{2}}{\psi ^{2}\parallel IL_{12} \parallel ^{2}_{\infty }}\right) ,\\\le & {} K_{6}(1+\mid Z \mid ^{2}). \end{aligned}$$

for condition:

$$\begin{aligned} \frac{a^{2}}{\psi ^{2}\parallel IL_{12} \parallel ^{2}_{\infty }}<1, \end{aligned}$$

where

$$\begin{aligned} K_{6}=4\parallel n^{'}\parallel ^{2}_{\infty }\left( \psi ^{2}\parallel IL_{12} \parallel ^{2}_{\infty }\right) . \end{aligned}$$

The linear growth criterion is therefore met.

We also confirm the Lipschitz criterion in the following way.

If

$$\begin{aligned}{} & {} \mid X_{1}(t,\,\,T_{1},\,\,C,\,\,D,\,\,IL_{2},\,\,IL_{12},\,\,Z)-X_{1}(t,\,\,T_{2},\,\,C,\,\,D,\,\,IL_{2},\,\,IL_{12},\,\,Z)\mid ^{2}\\{} & {} \quad =\mid n^{'}\big [(\alpha + \beta \alpha T_{2})+(-\alpha \beta T_{1}-\gamma - \phi C )\big ](T_{1}-T_{2})\mid ^{2},\\{} & {} \parallel X_{1}(t,\,\,T_{1},\,\,C,\,\,D,\,\,IL_{2},\,\,IL_{12},\,\,Z)-X_{1}(t,\,\,T_{2},\,\,C,\,\,D,\,\,IL_{2},\,\,IL_{12},\,\,Z)\parallel _{\infty }^{2}\\{} & {} \quad \le 2 \parallel n^{'}\parallel ^{2}_{\infty }\big (2(\alpha ^{2}+\alpha ^{2} \beta ^{2}\parallel T_{2}\parallel ^{2}_{\infty })+3(\alpha ^{2} \beta ^{2} \parallel T_{1}\parallel ^{2}_{\infty }+\gamma ^{2} \\{} & {} \quad + \phi ^{2} \parallel C \parallel ^{2}_{\infty } )\big ) \times \parallel T_{1}-T_{2}\parallel ^{2}_{\infty },\\{} & {} \parallel X_{1}(t,\,\,T_{1},\,\,C,\,\,D,\,\,IL_{2},\,\,IL_{12},\,\,Z)-X_{1}(t,\,\,T_{2},\,\,C,\,\,D,\,\,IL_{2},\,\,IL_{12},\,\,Z)\parallel _{\infty }^{2}\\{} & {} \quad \le \bar{K_{1}}\parallel T_{1}-T_{2}\parallel ^{2}_{\infty }, \end{aligned}$$

where

$$\begin{aligned} \bar{K_{1}}=2\parallel n^{'}\parallel ^{2}_{\infty }\left( 2(\alpha ^{2}+ \beta ^{2} \alpha ^{2} \parallel T_{2}\parallel ^{2}_{\infty })+3(\alpha ^{2} \beta ^{2} \parallel T_{1}\parallel ^{2}_{\infty }+\phi ^{2} \parallel C \parallel ^{2}_{\infty } + \gamma ^{2})\right) . \end{aligned}$$

If

$$\begin{aligned}{} & {} \mid X_{2}(t,\,\,T,\,\,C_{1},\,\,D,\,\,IL_{2},\,\,IL_{12},\,\,Z)-X_{2}(t,\,\,T,\,\,C_{2},\,\,D,\,\,IL_{2},\,\,IL_{12},\,\,Z)\mid ^{2}\\{} & {} \quad = \mid n^{'}(\phi C T+\rho C D-\kappa C)\mid ^{2},\\{} & {} \parallel X_{2}(t,\,\,T,\,\,C_{1},\,\,D,\,\,IL_{2},\,\,IL_{12},\,\,Z)-X_{2}(t,\,\,T,\,\,C_{2},\,\,D,\,\,IL_{2},\,\,IL_{12},\,\,Z)\parallel _{\infty }^{2}\\{} & {} \quad \le \parallel n^{'}\parallel ^{2}_{\infty }(3 \phi ^{2} \parallel T \parallel ^{2}_{\infty }+3\rho ^{2}\parallel D \parallel ^{2}_{\infty }+3\kappa ^{2})\parallel C_{1}-C_{2}\parallel ^{2}_{\infty },\\{} & {} \parallel X_{2}(t,\,\,T,\,\,C_{1},\,\,D,\,\,IL_{2},\,\,IL_{12},\,\,Z)-X_{2}(t,\,\,T,\,\,C_{2},\,\,D,\,\,IL_{2},\,\,IL_{12},\,\,Z)\parallel _{\infty }^{2}\\{} & {} \quad \le \bar{K_{2}}\parallel C_{1}-C_{2}\parallel ^{2}_{\infty }, \end{aligned}$$

where

$$\begin{aligned} \bar{K_{2}}=\parallel n^{'}\parallel ^{2}_{\infty }(3 \phi ^{2} \parallel T \parallel ^{2}_{\infty }+3\rho ^{2}\parallel D \parallel ^{2}_{\infty }+3\kappa ^{2}). \end{aligned}$$

If

$$\begin{aligned}{} & {} \mid X_{3}(t,\,\,T,\,\,C,\,\,D_{1},\,\,IL_{2},\,\,IL_{12},\,\,Z)-X_{3}(t,\,\,T,\,\,C,\,\,D_{2},\,\,IL_{2},\,\,IL_{12},\,\,Z)\mid ^{2}\\{} & {} \quad =\mid n^{'}\left( -\rho C -\omega -\lambda )\right) (D_{1}-D_{2})\mid ^{2},\\{} & {} \parallel X_{3}(t,\,\,T,\,\,C,\,\,D_{1},\,\,IL_{2},\,\,IL_{12},\,\,Z)-X_{3}(t,\,\,T,\,\,C,\,\,D_{2},\,\,IL_{2},\,\,IL_{12},\,\,Z)\parallel _{\infty }^{2}\\{} & {} \quad \le \parallel n^{'}\parallel ^{2}_{\infty }\bigg (3 \rho ^{2}\parallel C \parallel ^{2}_{\infty }+3\omega ^{2}+3\lambda ^{2})\bigg )\parallel D_{1}-D_{2}\parallel ^{2}_{\infty },\\{} & {} \parallel X_{3}(t,\,\,T,\,\,C,\,\,D_{1},\,\,IL_{2},\,\,IL_{12},\,\,Z)-X_{3}(t,\,\,T,\,\,C,\,\,D_{2},\,\,IL_{2},\,\,IL_{12},\,\,Z)\parallel _{\infty }^{2}\\{} & {} \quad \le \bar{K_{3}}\parallel D_{1}-D_{2}\parallel ^{2}_{\infty }, \end{aligned}$$

where

$$\begin{aligned} \bar{K_{3}}=\parallel n^{'}\parallel ^{2}_{\infty }\bigg (3(\parallel C \parallel ^{2}_{\infty } \rho ^{2}+\lambda ^{2}+\omega ^{2})\bigg ). \end{aligned}$$

If

$$\begin{aligned}{} & {} \mid X_{4}(t,\,\,T,\,\,C,\,\,D,\,\,{IL_{2}}_{1},\,\,IL_{12},\,\,Z)-X_{4}(t,\,\,T,\,\,C,\,\,D,\,\,{IL_{2}}_{2},\,\,IL_{12},\,\,Z)\mid ^{2}\\{} & {} \quad =\mid n^{'}(-d )({IL_{2}}_{1}-{IL_{2}}_{2})\mid ^{2},\\{} & {} \mid X_{4}(t,\,\,T,\,\,C,\,\,D,\,\,{IL_{2}}_{1},\,\,IL_{12},\,\,Z)-X_{4}(t,\,\,T,\,\,C,\,\,D,\,\,{IL_{2}}_{2},\,\,IL_{12},\,\,Z)\mid ^{2}\\{} & {} \quad =\mid n^{'}\mid ^{2}(d^{2})\mid {IL_{2}}_{1}-{IL_{2}}_{2}\mid ^{2},\\{} & {} \sup _{t\in D_{IL_{2}}}X_{4}(t,\,\,T,\,\,C,\,\,D,\,\,{IL_{2}}_{1},\,\,IL_{12},\,\,Z)-X_{4}(t,\,\,T,\,\,C,\,\,D,\,\,{IL_{2}}_{2},\,\,IL_{12},\,\,Z)\mid ^{2}\\{} & {} \quad =\sup _{t\in D_{n^{'}}}\mid n^{'}\mid ^{2}(d^{2})\sup _{t\in D_{IL_{2}}}\mid {IL_{2}}_{1}-{IL_{2}}_{2}\mid ^{2},\\{} & {} \parallel X_{4}(t,\,\,T,\,\,C,\,\,D,\,\,{IL_{2}}_{1},\,\,IL_{12},\,\,Z)-X_{4}(t,\,\,T,\,\,C,\,\,D,\,\,{IL_{2}}_{2},\,\,IL_{12},\,\,Z)\parallel _{\infty }^{2}\\{} & {} \quad \le \parallel n^{'}\parallel ^{2}_{\infty }(d^{2})\parallel {IL_{2}}_{1}-{IL_{2}}_{2}\parallel ^{2}_{\infty },\\{} & {} \parallel X_{4}(t,\,\,T,\,\,C,\,\,D,\,\,{IL_{2}}_{1},\,\,IL_{12},\,\,Z)-X_{4}(t,\,\,T,\,\,C,\,\,D,\,\,{IL_{2}}_{2},\,\,IL_{12},\,\,Z)\parallel _{\infty }^{2}\\{} & {} \quad \le \bar{K_{4}}\parallel {IL_{2}}_{1}-{IL_{2}}_{2}\parallel ^{2}_{\infty }, \end{aligned}$$

where

$$\begin{aligned} \bar{K_{4}}=\parallel n^{'}\parallel ^{2}_{\infty }(d^{2}). \end{aligned}$$

If

$$\begin{aligned}{} & {} \mid X_{5}(t,\,\,T,\,\,C,\,\,D,\,\,IL_{2},\,\,{IL_{12}}_{1},\,\,Z)-X_{5}(t,\,\,T,\,\,C,\,\,D,\,\,IL_{2},\,\,{IL_{12}}_{2},\,\,Z)\mid ^{2}\\{} & {} \quad =\mid n^{'}(-\psi )({IL_{12}}_{1}-{IL_{12}}_{2})\mid ^{2},\\{} & {} \mid X_{5}(t,\,\,T,\,\,C,\,\,D,\,\,IL_{2},\,\,{IL_{12}}_{1},\,\,Z)-X_{5}(t,\,\,T,\,\,C,\,\,D,\,\,IL_{2},\,\,{IL_{12}}_{2},\,\,Z)\mid ^{2}\\{} & {} \quad =\mid n^{'}\mid ^{2}(\psi ^{2})\mid {IL_{12}}_{1}-{IL_{12}}_{2}\mid ^{2},\\{} & {} \sup _{t\in D_{IL_{12}}}X_{5}(t,\,\,T,\,\,C,\,\,D,\,\,IL_{2},\,\,{IL_{12}}_{1},\,\,Z)-X_{5}(t,\,\,T,\,\,C,\,\,D,\,\,IL_{2},\,\,{IL_{12}}_{2},\,\,Z)\mid ^{2}\\{} & {} \quad =\sup _{t\in D_{n^{'}}}\mid n^{'}\mid ^{2}(\psi ^{2})\sup _{t\in D_{IL_{12}}}\mid {IL_{12}}_{1}-{IL_{12}}_{2}\mid ^{2},\\{} & {} \parallel X_{5}(t,\,\,T,\,\,C,\,\,D,\,\,IL_{2},\,\,{IL_{12}}_{1},\,\,Z)-X_{5}(t,\,\,T,\,\,C,\,\,D,\,\,IL_{2},\,\,{IL_{12}}_{2},\,\,Z)\parallel _{\infty }^{2}\\{} & {} \quad \le \parallel n^{'}\parallel ^{2}_{\infty }(\psi ^{2})\parallel {IL_{12}}_{1}-{IL_{12}}_{2}\parallel ^{2}_{\infty },\\{} & {} \parallel X_{5}(t,\,\,T,\,\,C,\,\,D,\,\,IL_{2},\,\,{IL_{12}}_{1},\,\,Z)-X_{5}(t,\,\,T,\,\,C,\,\,D,\,\,IL_{2},\,\,{IL_{12}}_{2},\,\,Z)\parallel _{\infty }^{2}\\{} & {} \quad \le \bar{K_{5}}\parallel {IL_{12}}_{1}-{IL_{12}}_{2}\parallel ^{2}_{\infty }, \end{aligned}$$

where

$$\begin{aligned} \bar{K_{5}}=\parallel n^{'}\parallel ^{2}_{\infty }(\psi ^{2}). \end{aligned}$$

If

$$\begin{aligned} \mid X_{6}(t,\,\,T,\,\,C,\,\,D,\,\,IL_{2},\,\,IL_{12},\,\,Z_{1})-X_{6}(t,\,\,T,\,\,C,\,\,D,\,\,IL_{2},\,\,IL_{12},\,\,Z_{2})\mid ^{2}= & {} \mid n^{'}(-a )(Z_{1}-Z_{2})\mid ^{2},\\ \mid X_{6}(t,\,\,T,\,\,C,\,\,D,\,\,IL_{2},\,\,IL_{12},\,\,Z_{1})-X_{6}(t,\,\,T,\,\,C,\,\,D,\,\,IL_{2},\,\,IL_{12},\,\,Z_{2})\mid ^{2}\le & {} \mid n^{'}\mid ^{2}a^{2}\mid (Z_{1}-Z_{2})\mid ^{2},\\ \sup _{t\in D_{Z}}\mid X_{6}(t,\,\,T,\,\,C,\,\,D,\,\,IL_{2},\,\,IL_{12},\,\,Z_{1})-X_{6}(t,\,\,T,\,\,C,\,\,D,\,\,IL_{2},\,\,IL_{12},\,\,Z_{2})\mid ^{2}\le & {} \sup _{t\in D_{n^{'}}}\mid n^{'}\mid ^{2}a^{2}\sup _{t\in D_{Z}}\mid Z_{1}-Z_{2}\mid ^{2},\\ \parallel X_{6}(t,\,\,T,\,\,C,\,\,D,\,\,IL_{2},\,\,IL_{12},\,\,Z_{1})-X_{6}(t,\,\,T,\,\,C,\,\,D,\,\,IL_{2},\,\,IL_{12},\,\,Z_{2})\parallel _{\infty }^{2}\le & {} \parallel n^{'}\parallel ^{2}_{\infty }a^{2}\parallel Z_{1}-Z_{2}\parallel _{\infty }^{2},\\ \parallel X_{6}(t,\,\,T,\,\,C,\,\,D,\,\,IL_{2},\,\,IL_{12},\,\,Z_{1})-X_{6}(t,\,\,T,\,\,C,\,\,D,\,\,IL_{2},\,\,IL_{12},\,\,Z_{2})\parallel _{\infty }^{2}\le & {} \bar{K_{6}}\parallel Z_{1}-Z_{2}\parallel ^{2}_{\infty }, \end{aligned}$$

involving

$$\begin{aligned} \bar{K_{6}}=\parallel n^{'}\parallel ^{2}_{\infty }a^{2}. \end{aligned}$$

Thus, with the following condition, there exists a unique solution for the system (3).

$$\begin{aligned} \max {\left\{ \begin{array}{ll} \frac{3}{\alpha ^{2}\parallel T \parallel ^{2}_{\infty }}\left( \alpha ^{2} \beta ^{2} \parallel T \parallel ^{2}_{\infty }+\gamma ^{2}+\phi ^{2} \parallel C \parallel ^{2}_{\infty } \right) ,\\ \frac{2(\phi ^{2}\parallel T \parallel ^{2}_{\infty }+\rho ^{2}\parallel D \parallel ^{2}_{\infty })}{\kappa ^{2}\parallel C \parallel ^{2}_{\infty }},\\ \frac{3}{\mu ^{2}}\left( \rho ^{2} \parallel C \parallel ^{2}_{\infty }+\omega ^{2} +\lambda ^{2}\right) ,\\ \frac{d^{2}}{\lambda ^{2}\parallel D \parallel ^{2}_{\infty }},\\ \frac{\psi ^{2}}{d^{2}\parallel IL_{2} \parallel ^{2}_{\infty }},\\ \frac{a^{2}}{\psi ^{2}\parallel IL_{12} \parallel ^{2}_{\infty }}. \end{array}\right. } < 1 \end{aligned}$$

## Solutions by fractal fractional operator

The newly developed model, stated in Eq. (3), will now have a numerical solution developed for it. Instead of using a traditional derivative operator in this instance, we employ an ML kernel.

$$\begin{aligned} {}^{FFM}_{0}D^{\xi ,\lambda }_{t} T(t)= & {} \alpha T (1-\beta T)-\gamma T- \phi C T,\\ {}^{FFM}_{0}D^{\xi ,\lambda }_{t} C(t)= & {} \phi C T+\rho C D-\kappa C,\\ {}^{FFM}_{0}D^{\xi ,\lambda }_{t} D(t)= & {} \mu -\rho C D-\omega D-\lambda D,\\ {}^{FFM}_{0}D^{\xi ,\lambda }_{t} IL_{2}(t)= & {} \lambda D-d IL_{2},\\ {}^{FFM}_{0}D^{\xi ,\lambda }_{t} IL_{12}(t)= & {} d IL_{2}-\psi IL_{12},\\ {}^{FFM}_{0}D^{\xi ,\lambda }_{t} Z(t)= & {} \psi IL_{12}-a Z. \end{aligned}$$

For clarity,

$$\begin{aligned} T_{1}(\,t, \,T, \,G)= & {} \,\alpha \,T (\,1\,-\,\beta \,T)- \phi C T-\gamma T,\\ C_{1}(\,t, \,T, \,G)= & {} \phi C T+\rho C D-\kappa C,\\ D_{1}(t, T, G)= & {} \mu -\rho C D-\omega D-\lambda D,\\ {IL_{2}}_{1}(t, S, G)= & {} \lambda D-d IL_{2},\\ {IL_{12}}_{1}(t, S, G)= & {} d IL_{2}-\psi IL_{12},\\ Z_{1}(t, T, G)= & {} \psi IL_{12}-a Z. \end{aligned}$$

We get the following outcomes by combining the ML kernel with the fractal-fractional integral.

4 $$\begin{aligned} T(t_{\varrho }+1)= & {} \frac{\lambda (1-\xi )}{A B(\xi )}t_{\varrho }^{\lambda -1}T_{1}(t_{\varrho },T(t_{\varrho }),G(t_{\varrho })))\nonumber \\{} & {} +\frac{\xi \lambda }{A B(\xi )\Gamma (\xi )}\sum ^{\varrho }_{\varpi =2}\int _{t_{\varpi }}^{t_{\varpi +1}}T_{1}(t,T,G)\upsilon ^{\xi -1}(t_{\varrho +1} -\upsilon )^{\xi -1}{d}\upsilon ,\nonumber \\ C(t_{\varrho }+1)= & {} \frac{\lambda (1-\xi )}{A B(\xi )}t_{\varrho }^{\lambda -1}C_{1}(t_{\varrho },T(t_{\varrho }),G(t_{\varrho })))\nonumber \\{} & {} +\frac{\xi \lambda }{A B(\xi )\Gamma (\xi )}\sum ^{\varrho }_{\varpi =2}\int _{t_{\varpi }}^{t_{\varpi +1}}C_{1}(t,T,G)\upsilon ^{\xi -1}(t_{\varrho +1} -\upsilon )^{\xi -1}{d}\upsilon ,\nonumber \\ D(t_{\varrho }+1)= & {} \frac{\lambda (1-\xi )}{A B(\xi )}t_{\varrho }^{\lambda -1}D_{1}(t_{\varrho },T(t_{\varrho }),G(t_{\varrho })))\nonumber \\{} & {} +\frac{\xi \lambda }{A B(\xi )\Gamma (\xi )}\sum ^{\varrho }_{\varpi =2}\int _{t_{\varpi }}^{t_{\varpi +1}}D_{1}(t,T,G)\upsilon ^{\xi -1}(t_{\varrho +1} -\upsilon )^{\xi -1}{d}\upsilon ,\nonumber \\ IL_{2}(t_{\varrho }+1)= & {} \frac{\lambda (1-\xi )}{A B(\xi )}t_{\varrho }^{\lambda -1}{IL_{2}}_{1}(t_{\varrho },T(t_{\varrho }),G(t_{\varrho })))\nonumber \\{} & {} +\frac{\xi \lambda }{A B(\xi )\Gamma (\xi )}\sum ^{\varrho }_{\varpi =2}\int _{t_{\varpi }}^{t_{\varpi +1}}{IL_{2}}_{1}(t,T,G)\upsilon ^{\xi -1}(t_{\varrho +1} -\upsilon )^{\xi -1}{d}\upsilon ,\nonumber \\ IL_{12}(t_{\varrho }+1)= & {} \frac{\lambda (1-\xi )}{A B(\xi )}t_{\varrho }^{\lambda -1}{IL_{12}}_{1}(t_{\varrho },T(t_{\varrho }),G(t_{\varrho })))\nonumber \\{} & {} +\frac{\xi \lambda }{A B(\xi )\Gamma (\xi )}\sum ^{\varrho }_{\varpi =2}\int _{t_{\varpi }}^{t_{\varpi +1}}{IL_{12}}_{1}(t,T,G)\upsilon ^{\xi -1}(t_{\varrho +1} -\upsilon )^{\xi -1}{d}\upsilon ,\nonumber \\ Z(t_{\varrho }+1)= & {} \frac{\lambda (1-\xi )}{A B(\xi )}t_{\varrho }^{\lambda -1}Z_{1}(t_{\varrho },T(t_{\varrho }),G(t_{\varrho })))\nonumber \\{} & {} +\frac{\xi \lambda }{A B(\xi )\Gamma (\xi )}\sum ^{\varrho }_{\varpi =2}\int _{t_{\varpi }}^{t_{\varpi +1}}Z_{1}(t,T,G)\upsilon ^{\xi -1}(t_{\varrho +1} -\upsilon )^{\xi -1}{d}\upsilon . \end{aligned}$$

where $$G(t_{\varrho })=C(t_{\varrho }),\,\, D(t_{\varrho }),\,\, IL_{2}(t_{\varrho }),\,\, IL_{12}(t_{\varrho }),\,\,Z(t_{\varrho })$$.

The system of Eq. (4) yields the following when the Newton polynomial is substituted.

5 $$\begin{aligned} \begin{aligned} T^{\varrho +1}&=\frac{\lambda (1-\xi )}{A B(\xi )}t_{\varrho }^{\lambda -1}T_{1}(t_{\varrho },T(t_{\varrho }),G(t_{\varrho }))+\frac{\xi \lambda }{A B(\xi )\Gamma (\xi )}\sum ^{\varrho }_{\varpi =2}S_{1} (t_{\varpi -2},T^{\varpi -2},G^{\varpi -2})\\&\times t^{\lambda -1}_{\varpi -2} \int _{t_{\varpi }}^{t_{\varpi +1}}(t_{\varrho +1}-\upsilon )^{\xi -1}{d}\upsilon +\frac{\xi \lambda }{A B(\xi )\Gamma (\xi )}\sum ^{\varrho }_{\varpi =2}\frac{1}{\Delta t}\Big [t^{\lambda -1}_{\varpi -1}T_{1}(t_{\varpi -1},T^{\varpi -1},G^{\varpi -1})\\&-t^{\lambda -1}_{\varpi -2} T_{1}(t_{\varpi -2},T^{\varpi -2},G^{\varpi -2})\Big ] \int _{t_{\varpi }}^{t_{\varpi +1}}(\upsilon -t_{\varpi -2})(t_{\varrho +1}-\upsilon )^{\xi -1}{d}\upsilon \\&+\frac{\xi }{A B(\xi )\Gamma (\xi )}\sum ^{\varrho }_{\varpi =2}\frac{1}{2\Delta t^{2}}\Big [t^{\lambda -1}_{\varpi }T_{1}(t_{\varpi },T^{\varpi },G^{\varpi }) -2t^{\lambda -1}_{\varpi -1}T_{1}(t_{\varpi -1},T^{\varpi -1},G^{\varpi -1})\\&+t^{\lambda -1}_{\varpi -2} T_{1}(t_{\varpi -2},T^{\varpi -2},G^{\varpi -2})\Big ] \int _{t_{\varpi }}^{t_{\varpi +1}}(\,\upsilon \,-\,t_{\,\varpi \,-\,2})(\,\upsilon \,-\,t_{\,\varpi \,-\,1})(t_{\varrho +1}-\upsilon )^{\xi -1}{d}\upsilon ,\\ C^{\varrho +1}&=\frac{\lambda (1-\xi )}{A B(\xi )}t_{\varrho }^{\lambda -1}C_{1}(t_{\varrho },T(t_{\varrho }),G(t_{\varrho }))+\frac{\xi \lambda }{A B(\xi )\Gamma (\xi )}\sum ^{\varrho }_{\varpi =2}C_{1} (t_{\varpi -2},T^{\varpi -2},G^{\varpi -2})\\&\times t^{\lambda -1}_{\varpi -2}\int _{t_{\varpi }}^{t_{\varpi +1}}(t_{\varrho +1}-\upsilon )^{\xi -1}{d}\upsilon \ +\frac{\xi \lambda }{A B(\xi )\Gamma (\xi )}\sum ^{\varrho }_{\varpi =2}\frac{1}{\Delta t}\Big [t^{\lambda -1}_{\varpi -1}C_{1}(t_{\varpi -1},T^{\varpi -1},G^{\varpi -1})\\&-t^{\lambda -1}_{\varpi -2} C_{1}(t_{\varpi -2},T^{\varpi -2},G^{\varpi -2})\Big ] \int _{t_{\varpi }}^{t_{\varpi +1}}(\upsilon -t_{\varpi -2})(t_{\varrho +1}-\upsilon )^{\xi -1}{d}\upsilon \\&+\frac{\xi \lambda }{A B(\xi )\Gamma (\xi )}\sum ^{\varrho }_{\varpi =2}\frac{1}{2\Delta t^{2}}\Big [t^{\lambda -1}_{\varpi } C_{1}(t_{\varpi },T^{\varpi },G^{\varpi }) -2t^{\lambda -1}_{\varpi -1}C_{1}(t_{\varpi -1},T^{\varpi -1},G^{\varpi -1})\\&+t^{\lambda -1}_{\varpi -2} C_{1}(t_{\varpi -2},T^{\varpi -2},G^{\varpi -2})\Big ] \int _{t_{\varpi }}^{t_{\varpi +1}}(\,\upsilon \,-\,t_{\,\varpi \,-\,2})(\,\upsilon \,-\,t_{\,\varpi \,-\,1})(t_{\varrho +1}-\upsilon )^{\xi -1}{d}\upsilon ,\\ D^{\varrho +1}&=\frac{\lambda (1-\xi )}{A B(\xi )}t_{\varrho }^{\lambda -1}I_{1}(t_{\varrho },T(t_{\varrho }),G(t_{\varrho }))+\frac{\xi \lambda }{A B(\xi )\Gamma (\xi )}\sum ^{\varrho }_{\varpi =2}D_{1} (t_{\varpi -2},T^{\varpi -2},G^{\varpi -2})\\&\times t^{\lambda -1}_{\varpi -2}\int _{t_{\varpi }}^{t_{\varpi +1}}(t_{\varrho +1}-\upsilon )^{\xi -1}{d}\upsilon +\frac{\xi \lambda }{A B(\xi )\Gamma (\xi )}\sum ^{\varrho }_{\varpi =2}\frac{1}{\Delta t} \Big [t^{\lambda -1}_{\varpi -1} D_{1}(t_{\varpi -1},T^{\varpi -1},G^{\varpi -1})\\&-t^{\lambda -1}_{\varpi -2} D_{1}(t_{\varpi -2},T^{\varpi -2},G^{\varpi -2})\Big ] \int _{t_{\varpi }}^{t_{\varpi +1}}(\upsilon -t_{\varpi -2})(t_{\varrho +1}-\upsilon )^{\xi -1}{d}\upsilon \\&+\frac{\xi \lambda }{A B(\xi )\Gamma (\xi )}\sum ^{\varrho }_{\varpi =2}\frac{1}{2\Delta t^{2}}\Big [t^{\lambda -1}_{\varpi }D_{1}(t_{\varpi },T^{\varpi },G^{\varpi }) -2t^{\lambda -1}_{\varpi -1}D_{1}(t_{\varpi -1},T^{\varpi -1},G^{\varpi -1})\\&+t^{\lambda -1}_{\varpi -2} D_{1}(t_{\varpi -2},T^{\varpi -2},G^{\varpi -2})\Big ] \int _{t_{\varpi }}^{t_{\varpi +1}}(\,\upsilon \,-\,t_{\,\varpi \,-\,2})(\,\upsilon \,-\,t_{\,\varpi \,-\,1})(t_{\varrho +1}-\upsilon )^{\xi -1}{d}\upsilon ,\\ IL_{2}^{\varrho +1}&=\frac{\lambda (1-\xi )}{A B(\xi )}t_{\varrho }^{\lambda -1}{IL_{2}}_{1}(t_{\varrho },T(t_{\varrho }),G(t_{\varrho }))+\frac{\xi \lambda }{A B(\xi )\Gamma (\xi )}\sum ^{\varrho }_{\varpi =2}{IL_{2}}_{1} (t_{\varpi -2},T^{\varpi -2},G^{\varpi -2})\\&\times t^{\lambda -1}_{\varpi -2}\int _{t_{\varpi }}^{t_{\varpi +1}}(t_{\varrho +1}-\upsilon )^{\xi -1}{d}\upsilon +\frac{\xi \lambda }{A B(\xi )\Gamma (\xi )}\sum ^{\varrho }_{\varpi =2}\frac{1}{\Delta t}\Big [t^{\lambda -1}_{\varpi -1} {IL_{2}}_{1}(t_{\varpi -1},T^{\varpi -1},G^{\varpi -1})\\&-t^{\lambda -1}_{\varpi -2} {IL_{2}}_{1}(t_{\varpi -2},T^{\varpi -2},G^{\varpi -2})\Big ] \int _{t_{\varpi }}^{t_{\varpi +1}}(\upsilon -t_{\varpi -2})(t_{\varrho +1}-\upsilon )^{\xi -1}{d}\upsilon \\&+\frac{\xi \lambda }{A B(\xi )\Gamma (\xi )}\sum ^{\varrho }_{\varpi =2}\frac{1}{2\Delta t^{2}}\Big [t^{\lambda -1}_{\varpi }{IL_{2}}_{1}(t_{\varpi },T^{\varpi },G^{\varpi }) -2t^{\lambda -1}_{\varpi -1}{IL_{2}}_{1}(t_{\varpi -1},T^{\varpi -1},G^{\varpi -1})\\&+ t^{\lambda -1}_{\varpi -2} {IL_{2}}_{1}(t_{\varpi -2},T^{\varpi -2},G^{\varpi -2})\Big ] \int _{t_{\varpi }}^{t_{\varpi +1}}(\,\upsilon \,-\,t_{\,\varpi \,-\,2})(\,\upsilon \,-\,t_{\,\varpi \,-\,1})(t_{\varrho +1}-\upsilon )^{\xi -1}{d}\upsilon ,\\ IL_{12}^{\varrho +1}&=\frac{\lambda (1-\xi )}{A B(\xi )}t_{\varrho }^{\lambda -1}{IL_{12}}_{1}(t_{\varrho },T(t_{\varrho }),G(t_{\varrho }))+\frac{\xi \lambda }{A B(\xi )\Gamma (\xi )}\sum ^{\varrho }_{\varpi =2}{IL_{12}}_{1} (t_{\varpi -2},T^{\varpi -2},G^{\varpi -2})\\&\times t^{\lambda -1}_{\varpi -2}\int _{t_{\varpi }}^{t_{\varpi +1}}(t_{\varrho +1}-\upsilon )^{\xi -1}{d}\upsilon +\frac{\xi \lambda }{A B(\xi )\Gamma (\xi )}\sum ^{\varrho }_{\varpi =2}\frac{1}{\Delta t}\Big [t^{\lambda -1}_{\varpi -1} {IL_{12}}_{1}(t_{\varpi -1},T^{\varpi -1},G^{\varpi -1})\\&-t^{\lambda -1}_{\varpi -2} {IL_{12}}_{1}(t_{\varpi -2},T^{\varpi -2},G^{\varpi -2})\Big ] \int _{t_{\varpi }}^{t_{\varpi +1}}(\upsilon -t_{\varpi -2})(t_{\varrho +1}-\upsilon )^{\xi -1}{d}\upsilon \\&+ \frac{\xi \lambda }{A B(\xi )\Gamma (\xi )}\sum ^{\varrho }_{\varpi =2}\frac{1}{2\Delta t^{2}}\Big [t^{\lambda -1}_{\varpi }{IL_{12}}_{1}(t_{\varpi },T^{\varpi },G^{\varpi }) -2t^{\lambda -1}_{\varpi -1}{IL_{12}}_{1}(t_{\varpi -1},T^{\varpi -1},G^{\varpi -1})\\&+ t^{\lambda -1}_{\varpi -2} {IL_{12}}_{1}(t_{\varpi -2},T^{\varpi -2},G^{\varpi -2})\Big ] \int _{t_{\varpi }}^{t_{\varpi +1}}(\,\upsilon \,-\,t_{\,\varpi \,-\,2})(\,\upsilon \,-\,t_{\,\varpi \,-\,1})(t_{\varrho +1}-\upsilon )^{\xi -1}{d}\upsilon ,\\ Z^{\varrho +1}&=\frac{\lambda (1-\xi )}{A B(\xi )}t_{\varrho }^{\lambda -1}Z_{1}(t_{\varrho },T(t_{\varrho }),G(t_{\varrho }))+\frac{\xi \lambda }{A B(\xi )\Gamma (\xi )}\sum ^{\varrho }_{\varpi =2}Z_{1} (t_{\varpi -2},T^{\varpi -2},G^{\varpi -2})\\&\times t^{\lambda -1}_{\varpi -2}\int _{t_{\varpi }}^{t_{\varpi +1}}(t_{\varrho +1} -\upsilon )^{\xi -1}{d}\upsilon \ +\frac{\xi \lambda }{A B(\xi )\Gamma (\xi )}\sum ^{\varrho }_{\varpi =2}\frac{1}{\Delta t}\Big [t^{\lambda -1}_{\varpi -1}Z_{1}(t_{\varpi -1},T^{\varpi -1},G^{\varpi -1})\\&- t^{\lambda -1}_{\varpi -2}Z_{1}(t_{\varpi -2},T^{\varpi -2},G^{\varpi -2})\Big ] \int _{t_{\varpi }}^{t_{\varpi +1}}(\upsilon -t_{\varpi -2})(t_{\varrho +1}-\upsilon )^{\xi -1}{d}\upsilon \\&+ \frac{\xi \lambda }{A B(\xi )\Gamma (\xi )}\sum ^{\varrho }_{\varpi =2}\frac{1}{2\Delta t^{2}}\Big [t^{\lambda -1}_{\varpi }Z_{1}(t_{\varpi },T^{\varpi },G^{\varpi }) -2t^{\lambda -1}_{\varpi -1}Z_{1}(t_{\varpi -1},T^{\varpi -1},G^{\varpi -1})\\&+t^{\lambda -1}_{\varpi -2} Z_{1}(t_{\varpi -2},T^{\varpi -2},G^{\varpi -2})\Big ] \int _{t_{\varpi }}^{t_{\varpi +1}}(\,\upsilon \,-\,t_{\,\varpi \,-\,2})(\,\upsilon \,-\,t_{\,\varpi \,-\,1})(t_{\varrho +1}-\upsilon )^{\xi -1}{d}\upsilon . \end{aligned} \end{aligned}$$

where $$G^{\varpi -2}=C^{\varpi -2},D^{\varpi -2},IL_{2}^{\varpi -2},IL_{12}^{\varpi -2},Z^{\varpi -2}$$,   $$G^{\varpi -1}=C^{\varpi -1},D^{\varpi -1},IL_{2}^{\varpi -1},IL_{12}^{\varpi -1},Z^{\varpi -1}$$,   $$G^{\varpi }=C^{\varpi },D^{\varpi },IL_{2}^{\varpi },IL_{12}^{\varpi },Z^{\varpi }$$, $$G(t_{\varrho })=C(t_{\varrho }), D(t_{\varrho }), IL_{2}(t_{\varrho }), IL_{12}(t_{\varrho }), Z(t_{\varrho })$$.

For the integral in Eq. (5), after making the computations and simplification, we get as follows:

$$\begin{aligned} T^{\varrho +1}= & {} \frac{\lambda (1-\xi )}{A B(\xi )}t_{\varrho }^{\lambda -1}T_{1}(t_{\varrho },T(t_{\varrho }),G(t_{\varrho })) +\frac{\xi \lambda (\Delta t)^{\xi }}{A B(\xi )\Gamma (\xi +1)}\sum ^{\varrho }_{\varpi =2} T_{1}(t_{\varpi -2},T^{\varpi -2},G^{\varpi -2})\\\times & {} t^{\lambda -1}_{\varpi -2} \Big [(\,\varrho \,-\,\varpi \,+\,1)^{\xi }-(\varrho -\varpi )^{\xi }\Big ]+\frac{\xi \lambda (\Delta t)^{\xi }}{A B(\xi )\Gamma (\xi +2)}\sum ^{\varrho }_{\varpi =2} \Big [t^{\lambda -1}_{\varpi -1}T_{1}(t_{\varpi -1},T^{\varpi -1},G^{\varpi -1})\\- & {} t^{\lambda -1}_{\varpi -2}T_{1}(t_{\varpi -2},T^{\varpi -2},G^{\varpi -2})\Big ] \Big [(\,\varrho \,-\,\varpi \,+\,1)^{\xi }(\varrho -\varpi +3+2\xi )-(\varrho -\varpi )^{\xi }(\varrho -\varpi +3+3\xi )\Big ]\\ {}+ & {} \frac{\xi \lambda (\Delta t)^{\xi }}{2A B(\xi )\Gamma (\xi +3)}\sum ^{\varrho }_{\varpi =2}\Big [t^{\lambda -1}_{\varpi }T_{1} (t_{\varpi },T^{\varpi },G^{\varpi }) -2t^{\lambda -1}_{\varpi -1}T_{1}(t_{\varpi -1},T^{\varpi -1},G^{\varpi -1})\\+ & {} t^{\lambda -1}_{\varpi -2}T_{1}(t_{\varpi -2},T^{\varpi -2},G^{\varpi -2})\Big ] \Big [(\,\varrho \,-\,\varpi \,+\,1)^{\xi }\Big \{2(\varrho -\varpi )^{2}+(10+3 \xi )(\varrho -\varpi )+12+9\xi +2\xi ^{2}\Big \}\\- & {} (\varrho -\varpi )^{\xi }\times \Big \{2(\varrho -\varpi )^{2}+(5\xi +10)(\varrho -\varpi )+12+18\xi +6\xi ^{2}\Big \}\Big ],\\ C^{\varrho +1}= & {} \frac{\lambda (1-\xi )}{A B(\xi )}t_{\varrho }^{\lambda -1}C_{1}(t_{\varrho },T(t_{\varrho }),G(t_{\varrho })) +\frac{\xi \lambda (\Delta t)^{\xi }}{A B(\xi )\Gamma (\xi +1)}\sum ^{\varrho }_{\varpi =2} C_{1}(t_{\varpi -2},T^{\varpi -2},G^{\varpi -2})\\ {}\times & {} t^{\lambda -1}_{\varpi -2} \Big [(\,\varrho \,-\,\varpi \,+\,1)^{\xi }-(\varrho -\varpi )^{\xi }\Big ]+\frac{\xi \lambda (\Delta t)^{\xi }}{A B(\xi )\Gamma (\xi +2)}\sum ^{\varrho }_{\varpi =2} \Big [t^{\lambda -1}_{\varpi -1}C_{1}(t_{\varpi -1},T^{\varpi -1},G^{\varpi -1})\\ {}- & {} t^{\lambda -1}_{\varpi -2}C_{1}(t_{\varpi -2},T^{\varpi -2},G^{\varpi -2})\Big ] \Big [(\,\varrho \,-\,\varpi \,+\,1)^{\xi }(\varrho -\varpi +3+2\xi )-(\varrho -\varpi )^{\xi }(\varrho -\varpi +3+3\xi )\Big ]\\+ & {} \frac{\xi \lambda (\Delta t)^{\xi }}{2A B(\xi )\Gamma (\xi +3)}\sum ^{\varrho }_{\varpi =2}\Big [t^{\lambda -1}_{\varpi }C_{1} (t_{\varpi },T^{\varpi },G^{\varpi }) -2t^{\lambda -1}_{\varpi -1}C_{1}(t_{\varpi -1},T^{\varpi -1},G^{\varpi -1})\\+ & {} t^{\lambda -1}_{\varpi -2}C_{1}(t_{\varpi -2},T^{\varpi -2},G^{\varpi -2})\Big ] \Big [(\,\varrho \,-\,\varpi \,+\,1)^{\xi }\Big \{2(\varrho -\varpi )^{2}+(10+3 \xi )(\varrho -\varpi )+12+9\xi +2\xi ^{2}\Big \}\\- & {} (\varrho -\varpi )^{\xi }\times \Big \{2(\varrho -\varpi )^{2}+(5\xi +10)(\varrho -\varpi )+12+18\xi +6\xi ^{2}\Big \}\Big ], \end{aligned}$$

$$\begin{aligned} D^{\varrho +1}= & {} \frac{\lambda (1-\xi )}{A B(\xi )}t_{\varrho }^{\lambda -1}D_{1}(t_{\varrho },T(t_{\varrho }),G(t_{\varrho }))+\frac{\xi \lambda (\Delta t)^{\xi }}{A B(\xi )\Gamma (\xi +1)}\sum ^{\varrho }_{\varpi =2} D_{1}(t_{\varpi -2},T^{\varpi -2},G^{\varpi -2})\\ {}\times & {} t^{\lambda -1}_{\varpi -2} \Big [(\,\varrho \,-\,\varpi \,+\,1)^{\xi }-(\varrho -\varpi )^{\xi }\Big ]+\frac{\xi \lambda (\Delta t)^{\xi }}{A B(\xi )\Gamma (\xi +2)}\sum ^{\varrho }_{\varpi =2} \Big [t^{\lambda -1}_{\varpi -1}D_{1}(t_{\varpi -1},T^{\varpi -1},G^{\varpi -1})\\- & {} t^{\lambda -1}_{\varpi -2}D_{1}(t_{\varpi -2},T^{\varpi -2},G^{\varpi -2})\Big ] \Big [(\,\varrho \,-\,\varpi \,+\,1)^{\xi }(\varrho -\varpi +3+2\xi )-(\varrho -\varpi )^{\xi }(\varrho -\varpi +3+3\xi )\Big ]\\+ & {} \frac{\xi \lambda (\Delta t)^{\xi }}{2A B(\xi )\Gamma (\xi +3)}\sum ^{\varrho }_{\varpi =2}\Big [t^{\lambda -1}_{\varpi }D_{1} (t_{\varpi },T^{\varpi },G^{\varpi }) -2t^{\lambda -1}_{\varpi -1}D_{1}(t_{\varpi -1},T^{\varpi -1},G^{\varpi -1})\\+ & {} t^{\lambda -1}_{\varpi -2}D_{1}(t_{\varpi -2},T^{\varpi -2},G^{\varpi -2})\Big ] \Big [(\,\varrho \,-\,\varpi \,+\,1)^{\xi }\Big \{2(\varrho -\varpi )^{2}+(10+3 \xi )(\varrho -\varpi )+12+9\xi +2\xi ^{2}\Big \}\\- & {} (\varrho -\varpi )^{\xi }\times \Big \{2(\varrho -\varpi )^{2}+(5\xi +10)(\varrho -\varpi )+12+18\xi +6\xi ^{2}\Big \}\Big ],\\ IL_{2}^{\varrho +1}= & {} \frac{\lambda (1-\xi )}{A B(\xi )}t_{\varrho }^{\lambda -1}{IL_{2}}_{1}(t_{\varrho },T(t_{\varrho }),G(t_{\varrho })) +\frac{\xi \lambda (\Delta t)^{\xi }}{A B(\xi )\Gamma (\xi +1)}\sum ^{\varrho }_{\varpi =2} {IL_{2}}_{1}(t_{\varpi -2},T^{\varpi -2},G^{\varpi -2})\\\times & {} t^{\lambda -1}_{\varpi -2} \Big [(\,\varrho \,-\,\varpi \,+\,1)^{\xi }-(\varrho -\varpi )^{\xi }\Big ]+\frac{\xi \lambda (\Delta t)^{\xi }}{A B(\xi )\Gamma (\xi +2)}\sum ^{\varrho }_{\varpi =2} \Big [t^{\lambda -1}_{\varpi -1}{IL_{2}}_{1}(t_{\varpi -1},T^{\varpi -1},G^{\varpi -1})\\- & {} t^{\lambda -1}_{\varpi -2}{IL_{2}}_{1}(t_{\varpi -2},T^{\varpi -2},G^{\varpi -2})\Big ] \Big [(\,\varrho \,-\,\varpi \,+\,1)^{\xi }(\varrho -\varpi +3+2\xi )-(\varrho -\varpi )^{\xi }(\varrho -\varpi +3+3\xi )\Big ]\\ {}+ & {} \frac{\xi \lambda (\Delta t)^{\xi }}{2A B(\xi )\Gamma (\xi +3)}\sum ^{\varrho }_{\varpi =2}\Big [t^{\lambda -1}_{\varpi }{IL_{2}}_{1} (t_{\varpi },T^{\varpi },G^{\varpi }) -2t^{\lambda -1}_{\varpi -1}{IL_{2}}_{1}(t_{\varpi -1},T^{\varpi -1},G^{\varpi -1})\\+ & {} t^{\lambda -1}_{\varpi -2}{IL_{2}}_{1}(t_{\varpi -2},T^{\varpi -2},G^{\varpi -2})\Big ] \Big [(\,\varrho \,-\,\varpi \,+\,1)^{\xi }\Big \{2(\varrho -\varpi )^{2}+(10+3 \xi )(\varrho -\varpi )+12+9\xi +2\xi ^{2}\Big \}\\- & {} (\varrho -\varpi )^{\xi }\times \Big \{2(\varrho -\varpi )^{2}+(5\xi +10)(\varrho -\varpi )+12+18\xi +6\xi ^{2}\Big \}\Big ],\\ IL_{12}^{\varrho +1}= & {} \frac{\lambda (1-\xi )}{A B(\xi )}t_{\varrho }^{\lambda -1}{IL_{12}}_{1}(t_{\varrho },T(t_{\varrho }),G(t_{\varrho })) +\frac{\xi \lambda (\Delta t)^{\xi }}{A B(\xi )\Gamma (\xi +1)}\sum ^{\varrho }_{\varpi =2} {IL_{12}}_{1}(t_{\varpi -2},T^{\varpi -2},G^{\varpi -2})\\ {}\times & {} t^{\lambda -1}_{\varpi -2} \Big [(\,\varrho \,-\,\varpi \,+\,1)^{\xi }-(\varrho -\varpi )^{\xi }\Big ]+\frac{\xi \lambda (\Delta t)^{\xi }}{A B(\xi )\Gamma (\xi +2)}\sum ^{\varrho }_{\varpi =2} \Big [t^{\lambda -1}_{\varpi -1}{IL_{12}}_{1}(t_{\varpi -1},T^{\varpi -1},G^{\varpi -1})\\- & {} t^{\lambda -1}_{\varpi -2}{IL_{12}}_{1}(t_{\varpi -2},T^{\varpi -2},G^{\varpi -2})\Big ] \Big [(\,\varrho \,-\,\varpi \,+\,1)^{\xi }(\varrho -\varpi +3+2\xi )-(\varrho -\varpi )^{\xi }(\varrho -\varpi +3+3\xi )\Big ]\\+ & {} \frac{\xi \lambda (\Delta t)^{\xi }}{2A B(\xi )\Gamma (\xi +3)}\sum ^{\varrho }_{\varpi =2}\Big [t^{\lambda -1}_{\varpi }{IL_{12}}_{1} (t_{\varpi },T^{\varpi },G^{\varpi }) -2t^{\lambda -1}_{\varpi -1}{IL_{12}}_{1}(t_{\varpi -1},T^{\varpi -1},G^{\varpi -1})\\+ & {} t^{\lambda -1}_{\varpi -2}{IL_{12}}_{1}(t_{\varpi -2},T^{\varpi -2},G^{\varpi -2})\Big ] \Big [(\,\varrho \,-\,\varpi \,+\,1)^{\xi }\Big \{2(\varrho -\varpi )^{2}+(10+3 \xi )(\varrho -\varpi )+12+9\xi +2\xi ^{2}\Big \}\\- & {} (\varrho -\varpi )^{\xi }\times \Big \{2(\varrho -\varpi )^{2}+(5\xi +10)(\varrho -\varpi )+12+18\xi +6\xi ^{2}\Big \}\Big ],\\ Z^{\varrho +1}= & {} \frac{\lambda (1-\xi )}{A B(\xi )}t_{\varrho }^{\lambda -1}Z_{1}(t_{\varrho },T(t_{\varrho }),G(t_{\varrho }))+\frac{\xi \lambda (\Delta t)^{\xi }}{A B(\xi )\Gamma (\xi +1)}\sum ^{\varrho }_{\varpi =2} Z_{1}(t_{\varpi -2},T^{\varpi -2},G^{\varpi -2})\\ {}\times & {} t^{\lambda -1}_{\varpi -2} \Big [(\,\varrho \,-\,\varpi \,+\,1)^{\xi }-(\varrho -\varpi )^{\xi }\Big ]+\frac{\xi \lambda (\Delta t)^{\xi }}{A B(\xi )\Gamma (\xi +2)}\sum ^{\varrho }_{\varpi =2} \Big [t^{\lambda -1}_{\varpi -1}Z_{1}(t_{\varpi -1},T^{\varpi -1},G^{\varpi -1})\\ {}- & {} t^{\lambda -1}_{\varpi -2}Z_{1}(t_{\varpi -2},T^{\varpi -2},G^{\varpi -2})\Big ] \Big [(\,\varrho \,-\,\varpi \,+\,1)^{\xi }(\varrho -\varpi +3+2\xi )-(\varrho -\varpi )^{\xi }(\varrho -\varpi +3+3\xi )\Big ]\\+ & {} \frac{\xi \lambda (\Delta t)^{\xi }}{2A B(\xi )\Gamma (\xi +3)}\sum ^{\varrho }_{\varpi =2}\Big [t^{\lambda -1}_{\varpi }Z_{1} (t_{\varpi },T^{\varpi },G^{\varpi }) -2t^{\lambda -1}_{\varpi -1}Z_{1}(t_{\varpi -1},T^{\varpi -1},G^{\varpi -1})\\+ & {} t^{\lambda -1}_{\varpi -2}Z_{1}(t_{\varpi -2},T^{\varpi -2},G^{\varpi -2})\Big ] \Big [(\,\varrho \,-\,\varpi \,+\,1)^{\xi }\Big \{2(\varrho -\varpi )^{2}+(10+3 \xi )(\varrho -\varpi )+12+9\xi +2\xi ^{2}\Big \}\\- & {} (\varrho -\varpi )^{\xi }\times \Big \{2(\varrho -\varpi )^{2}+(5\xi +10)(\varrho -\varpi )+12+18\xi +6\xi ^{2}\Big \}\Big ]. \end{aligned}$$

## Simulation explanation

**Figure 3:**
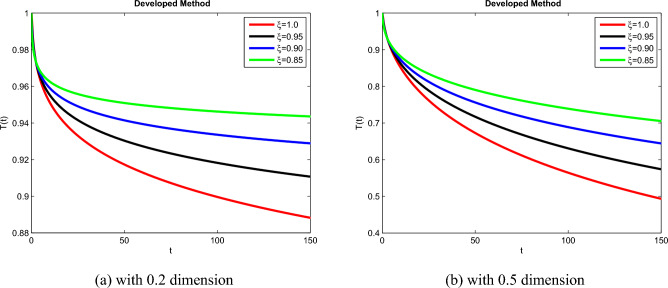
Simulation of *T*(*t*) using FFM with different dimensions.

**Figure 4:**
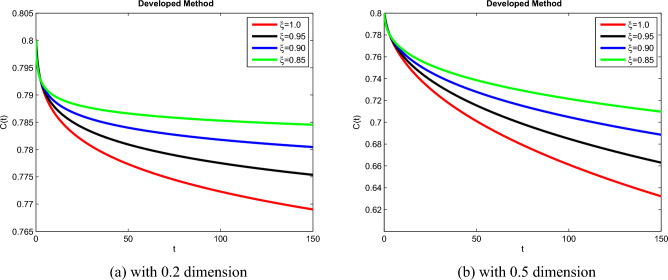
Simulation of *C*(*t*) using FFM with different dimensions.

**Figure 5:**
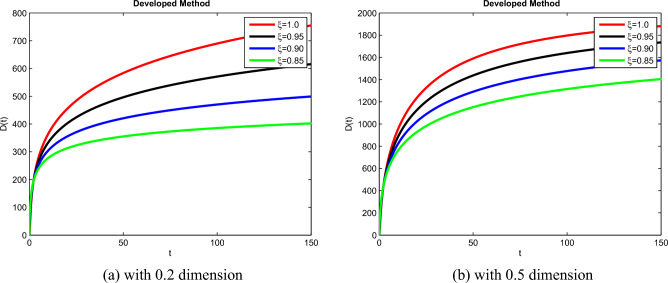
Simulation of *D*(*t*) using FFM with different dimensions.

**Figure 6:**
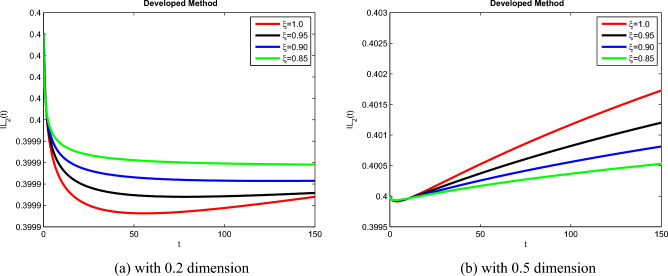
Simulation of $IL_{2}(t)$ using FFM with different dimensions.

**Figure 7:**
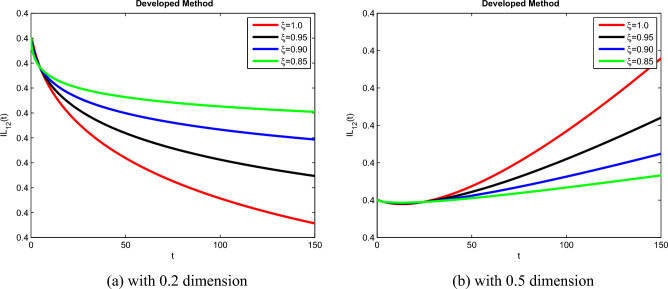
Simulation of $IL_{12}(t)$ using FFM with different dimensions.

**Figure 8:**
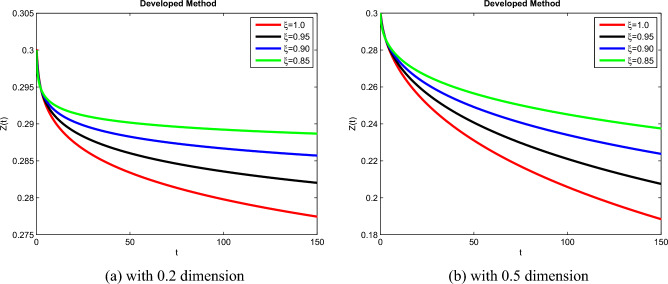
Simulation of *Z*(*t*) using FFM with different dimensions.

The theoretical results and their efficacy are investigated using the advanced approach. We do a simulation analysis of the recently created system TCD$IL_{2} IL_{12}$Z. Using non-integer parametric variables in the lung cancer model has allowed us to arrive at some fascinating results. We may get dependable answers by decreasing fractional values for the individuals $T(t), C(t), D(t), IL_{2}(t), IL_{12}(t)$, and *Z*(*t*) in Figs. 3, 4, 5, 6, 7 and 8. Approximate answers for the lung cancer model are found using MATLAB code. $T(0)=1.0, C(0)=0.8, D(0)=0.3, IL_{2}(0)=0.4, IL_{12}(0)=0.4$, and $Z(0)=0.3$ are the starting conditions employed in the recently constructed model. The parameters that were employed in the system that was built are $\beta =0.00000000102, \alpha =0.0514,\phi =0.0000001, \gamma =0.1, \mu =480, \kappa =0.02, \omega =0.24, \rho =0.00000001, \psi =0.0003, \lambda =0.0000002, a=0.04$, and $d=0.0003$.

The dynamics of tumor cell *T*, cancer cell *C*, $IL_{2}$ cytokine, $IL_{12}$ cytokine, and *Z* anti-PD-L1 inhibitor are shown in Figures 3, 4, 6, 7, and 8. During this time, all of the compartments sharply decrease before approaching their stable positions using various dimensions. The dynamics of dendritic cells *D* are shown in Fig. 5, where the number of individuals grows fast and, with the passage of time, each compartment achieves its stable location utilizing a distinct dimension. Cytokines and anti-PD-L1 inhibitors are seen to cause a dramatic drop in cancer cells; this can be seen in Figs. 4, 6, 7, and 8 using various dimensions. A comparison of dimensions 0.2 and 0.5 shows similar results with little effects; however, reducing dimensions yields more acceptable results, as shown in Fig. 8. Furthermore, anti-PD-L1 cells and cytokines have been shown to support the immune system by boosting the generation of CD4+T and CD8+T lymphocytes and reducing the number of cancer cells. Along with aiding in the creation of cells that the body’s cancer cells kill, anti-PD-L1 also aids in the reduction of cancerous cells. It foretells what this study will uncover in the future and how we will be able to more effectively lower the amount of cancer cells that spread throughout the body. It can be deduced from Figs. 5 and 6 that the combine measures of dendritic cells and cytokine IL2 need to increase as it play the key role in the activation of antigen-specific CD8+T lymphocytes to improve the low immune individuals which is initiative step to fight against cancer cells while cytokine IL2 directly boost the immune system. Similarly, the anti-PD-L1 inhibitor rises gradually which directly helps to kill PD-L1 cells and cancer cells. It provide us better control by taking combine measures to boost immune system and killing lung cancer cells in the body which can be seen in Figs. 5, 6,7 and 8 respectively. It is observed that, when we compare to the traditional derivative, the FFM technique produces superior results for every sub-compartment by decreasing fractional values and its dimensions. It is also mentioned that as dimensions and fractional values are decreased, the solutions for every compartment become more reliable and exact for control purpose.

## Conclusion

This paper formulates a fractional order TCD$IL_{2} IL_{12}$Z lung cancer model by adding cytokines and an anti-PD-L1 inhibitor to strengthen the immune system in those with weakened immune systems. By injecting anti-cancer cells, which strengthen people’s immune systems and eradicate sickness from the environment, we show how to prevent the spread of illness. The harmful lung cancer illness is studied along with prevention and therapy options to assess the true global effect of lung cancer. To do this, a quantitative and qualitative investigation of the generated system is evaluated to confirm its stable state for a continuous dynamical system. In order to understand the dynamics of the epidemic, it is necessary to know how the model behaves under constrained conditions, which is determined by local stability analysis. We also confirm that there exist bounded and unique solutions for the fractional order lung cancer disease model. We confirm its existence and analyze the effects of international efforts to slow the lung cancer disease’s spread. In order to evaluate the overall effect of cytokines and anti-PD-L1 inhibitor for persons with weakened immune systems, the model is examined for global stability using Lyapunov functions with first derivative. It has been demonstrated that cytokines and anti-PD-L1 inhibitor therapies for patients with compromised immune systems reduce the number of cancer cases. Using various fractional values and dependable, practical results, the Fractal-Fractional Operator (FFO) is utilized to continuously track the disease’s progress. After including cytokines and anti-PD-L1 inhibitor measures, we use MATLAB for numerical simulation to observe the control of the lung cancer disease in the community. Predictions for future research will help to comprehend the behavior and environmental spread of lung cancer sickness as well as the early detection process may also be made based on our well-founded findings. In future the developed lung cancer model can be tested on any specific reginal real data and will be helpful control strategies. Also optimal control strategies can also be applied, it may provide better control under modified fractional operator. As its newly developed system, so fuzzy and optimization approach can be unutilized to investigate lung cancer in different aspects.

## Data Availability

All data generated or analysed during this study are included in this published article.
